# Osteogenic‐Like Microenvironment of Renal Interstitium Induced by Osteomodulin Contributes to Randall's Plaque Formation

**DOI:** 10.1002/advs.202405875

**Published:** 2024-09-03

**Authors:** Zewu Zhu, Fang Huang, Meng Gao, Minghui Liu, Youjie Zhang, Liang Tang, Jian Wu, Hao Yu, Cheng He, Jinbo Chen, Zhongqing Yang, Zhiyong Chen, Yang Li, Hequn Chen, Ting Lei, Feng Zeng, Yu Cui

**Affiliations:** ^1^ Department of Urology Xiangya Hospital Central South University Changsha Hunan 410008 China; ^2^ National Clinical Research Center for Geriatric Disorders Xiangya Hospital Central South University Changsha Hunan 410008 China; ^3^ Department of Internal Medicine Section Endocrinology Yale University School of Medicine New Haven CT 06519 USA; ^4^ Department of Orthopedics Xiangya Hospital Central South University Changsha Hunan 410008 China; ^5^ Department of Orthopaedic Surgery The First Affiliated Hospital School of Medicine Zhejiang University Hangzhou Zhejiang 310006 China

**Keywords:** biomineralization, fibroblasts, kidney stones, osteomodulin, Randall's plaques

## Abstract

Calcium oxalate (CaOx) kidney stones are common and recurrent, lacking pharmacological prevention. Randall's plaques (RPs), calcium deposits in renal papillae, serve as niduses for some CaOx stones. This study explores the role of osteogenic‐like cells in RP formation resembling ossification. CaP crystals deposit around renal tubules, interstitium, and blood vessels in RP tissues. Human renal interstitial fibroblasts (hRIFs) exhibit the highest osteogenic‐like differentiation potential compared to chloride voltage‐gated channel Ka positive tubular epithelial cells, aquaporin 2 positive collecting duct cells, and vascular endothelial cells, echoing the upregulated osteogenic markers primarily in hRIFs within RP tissues. Utilizing RNA‐seq, osteomodulin (OMD) is found to be upregulated in hRIFs within RP tissues and hRIFs following osteogenic induction. Furthermore, OMD colocalizes with CaP crystals and calcium vesicles within RP tissues. OMD can enhance osteogenic‐like differentiation of hRIFs in vitro and in vivo. Additionally, crystal deposits are attenuated in mice with *Omd* deletion in renal interstitial fibroblasts following CaOx nephrocalcinosis induction. Mechanically, a positive feedback loop of OMD/BMP2/BMPR1A/RUNX2/OMD drives hRIFs to adopt osteogenic‐like fates, by which OMD induces osteogenic‐like microenvironment of renal interstitium to participate in RP formation. We identify OMD upregulation as a pathological feature of RP, paving the way for preventing CaOx stones.

## Graphical abstract

Randall's plaques, calcium deposits in renal papillae, serve as a nidus for some calcium oxalate (CaOx) kidney stones. This study finds a positive feedback loop of OMD/BMP2/BMPR1A/RUNX2/OMD driving renal interstitial fibroblasts to adopt osteogenic‐like fates, by which OMD participates in Randall's plaque formation. It paves a new way for developing preventive medications for CaOx stones.

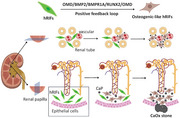

## 1 Introduction

Nephrolithiasis is a common urological disease,^[^ ^1^ ^]^ leading to pain, urinary tract infections, and even urosepsis as well as kidney loss.^[^ ^2^ ^]^ The prevalence of nephrolithiasis has significantly increased in the last three decades, afflicting ≈10% of the population worldwide.^[^ ^1^, ^3^ ^]^ Calcium oxalate (CaOx) accounts for more than 80% of kidney stones, with a recurrence rate of 50% within 5–10 years.^[^ ^4^ ^]^ However, there has been a lack of breakthroughs in understanding the pathophysiology and developing effective prophylaxis for CaOx stones.^[^ ^5^ ^]^

Randall's plaques (RPs), reported by Randall in 1937,^[^ ^6^ ^]^ originate from calcium phosphate (CaP) crystal deposits within the renal interstitium, grow outward, and eventually breach the renal papillary surface, serving as an attachment for CaOx crystals to develop at least some idiopathic CaOx stones.^[^ ^7^, ^8^ ^]^ Increasing evidence supported that RP formation shared similarities with the process of ossification. Nanoscale analysis of RP showed calcified organic vesicles and nanometric mineral granules primarily consisting of CaP, with carbonate present in the core.^[^ ^9^, ^10^ ^]^ Multiple osteogenesis‐associated proteins were upregulated in RP tissues, such as osteopotin,^[^ ^11^, ^12^, ^13^ ^]^ bone morphogenetic protein 2 (BMP2),^[^ ^14^, ^15^ ^]^ and collagen.^[^ ^16^ ^]^ Moreover, a spatially anchored transcriptomic atlas of RP highlighted the important role of osteogenic‐like cells surrounding mineralization areas.^[^ ^17^ ^]^ Human proximal renal tubular epithelial cells (HK‐2) and human renal interstitial fibroblasts (hRIFs) adopted osteogenic‐like phenotypes to form CaP nodules following osteogenic induction.^[^ ^18^, ^19^ ^]^ Despite significant progresses in unraveling the structure of RPs and identifying cells with potential for osteogenic‐like differentiation,^[^ ^20^ ^]^ it remains unclear that the dominant cell type and its potential molecular mechanisms drive the osteogenic‐like microenvironment of renal interstitium.

In this work, we procured difficult‐to‐obtain renal papilla specimens from patients with CaOx stones undergoing nephrectomy due to renal cancers, and revealed that calcium deposits were distributed around renal tubules, renal interstitium, and peritubular blood vessels in RP tissues. Based on the location of calcium deposits identified in our study and others,^[^ ^21^, ^22^, ^23^, ^24^ ^]^ we evaluated the potential of osteogenic‐like differentiation in vitro of primary human tubular epithelial cells, primary human collecting duct cells, hRIFs, and primary human renal peritubular capillary endothelial cells (hRPCECs). hRIFs were identified to have the highest potential of osteogenic‐like differentiation among these cells. Next, we utilized high‐throughput transcriptome sequencing of RP tissues and osteogenic‐induced hRIFs to explore the potentially pivotal regulators. Subsequently, osteomodulin (OMD) was identified as a potential molecular marker for ectopic calcification in renal interstitium, which drove the osteogenic‐like differentiation of hRIFs and induced osteogenic‐like microenvironment of renal interstitium to participate in RP formation.

## 2 Results

### 2.1 RPs Served as Nidus for Some CaOx Stones

**Figure 1:**
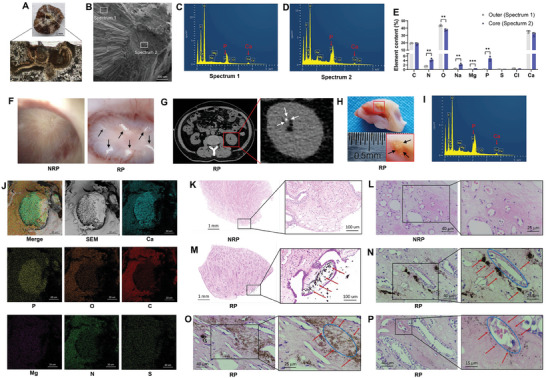
CaOx stone‐related Randall's plaques (RPs) originated from calcium phosphate (CaP) deposits within renal papillae. A) The structure of kidney CaOx stones examined by polarized light microscopy. B–E) Scanning electron microscopy (SEM) coupled with X‐ray energy dispersive spectroscopy (EDS) determined the elemental component of the core and outer regions in CaOx stones (*n* = 3). The voltage of EDS was set to 5 kV. F) Representative images of normal renal papillae (NRP) and RP captured by ureteroscopy. G) A noncontrast CT scan showed a renal papilla with RP (white arrows) in a patient with renal cancer. H) An intact renal papilla with RP (black arrows) obtained from a patient with renal cancer undergoing nephrectomy. I,J) SEM showed that RP breached the mucosa of the renal papilla, and EDS determined the elemental component of RP. K,L) Von‐Kossa staining of NRP. (M–P) Von‐Kossa staining of RP, with calcium deposits around renal tubules (N), renal interstitium (O), and peritubular blood vessels (P). Blue circles and red arrows indicated the location of calcium deposits.

Polarized light microscopy showed that CaOx stones had a regularly structured circular pattern in the core region, gradually transitioning into an irregularly stacked crystal structure (**Figure** **1**A). Consistently, scanning electron microscopy (SEM) coupled with energy‐dispersive spectrometry (EDS) indicated that the core region of CaOx stones contained calcium phosphate (CaP) (Figure 1B–E). Renal papilla with RP (Figure 1F) was obtained from patients receiving nephrectomy due to renal cancers (Figure 1G,H). We utilized SEM to illustrate that RP breached the mucosa of the renal papilla, and the composition of RP echoed that of the core region in CaOx stones (Figure 1I,J). Moreover, Von‐Kossa staining (Figure 1K–P) further showed that calcium deposits were distributed around renal tubules (Figure 1N), renal interstitium (Figure 1O), and peritubular blood vessels (Figure 1P) in RP tissues.

### 2.2 hRIFs Adopt Osteogenic‐Like Phenotypes in RP Tissues

**Figure 2:**
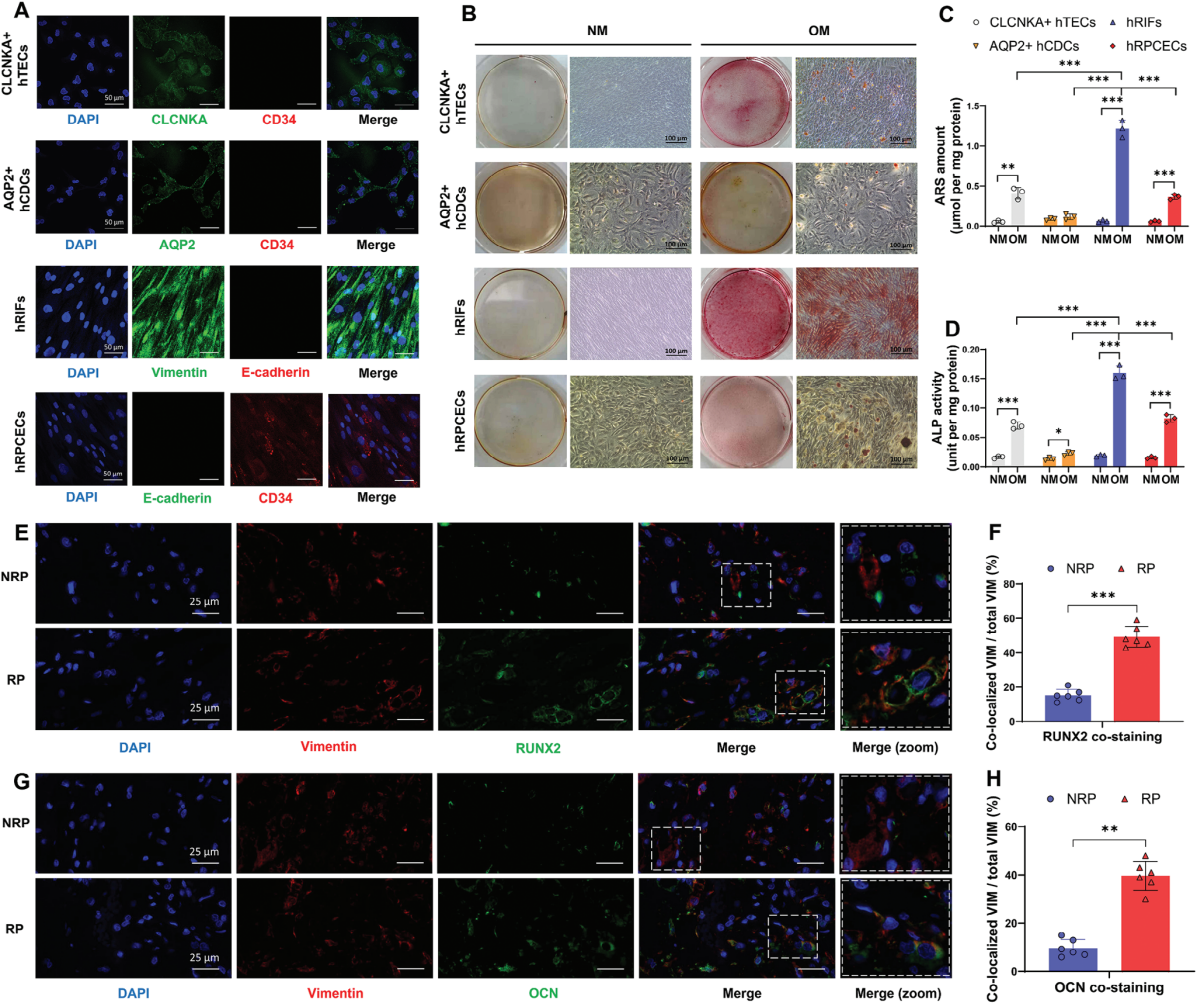
hRIFs adopted osteogenic‐like fates in RP tissues. A) The isolated human CLCNKA positive tubular epithelial cells (CLCNKA+ hTECs) were identified by immunofluorescence staining for CLCNKA and CD34. The isolated human AQP2 positive collecting duct cells (AQP2+ hCDCs) were identified by immunofluorescence staining for AQP2 and CD34. The isolated hRIFs were identified by immunofluorescence staining for VIM and E‐cadherin. The isolated human renal peritubular capillary endothelial cells (hRPCECs) were identified by immunofluorescence staining for CD34 and E‐cadherin. CLCNKA, chloride voltage‐gated channel Ka; AQP2, aquaporin 2; VIM, vimentin. B) Alizarin red staining for CLCNKA+ hTECs, AQP2+ hCDCs, hRIFs, and hRPCECs cultured in normal medium (NM) or osteogenic medium (OM) for 14 days; *n* = 3. C) Alizarin red in cell layers was quantified and normalized to the total protein of cell lysate, expressed as µmol per mg of protein; *n* = 3. D) Alkaline phosphatase (ALP) activity of CLCNKA+ hTECs, AQP2+ hCDCs, hRIFs, and hRPCECs cultured in NM or OM for 14 days. ALP activity was normalized to the total protein of cell lysate, expressed as unit per mg of protein; *n* = 3. E–H) Immunofluorescence costaining for VIM and RUNX2 or OCN in NRP (*n* = 6) and RP (*n* = 6) tissues. The percentage of the colocalized VIM to the total VIM was calculated to semiquantify the expression of RUNX2 or OCN in hRIFs.

Our previous studies and others have elucidated that the RP formation involved a multistep process characterized by the deposition of CaP mixed with an organic matrix, wherein osteogenic‐like cells play a pivotal role.^[^ ^19^, ^25^ ^]^ Previous studies found calcium deposits in the vicinity of the thin ascending limb of Henle's loop identified by chloride voltage‐gated channel Ka (CLCNKA) staining^[^ ^21^ ^]^ and the inner medullary collecting ducts identified by aquaporin 2 (AQP2) staining,^[^ ^22^ ^]^ and we found calcium deposits in the renal interstitium and in the vicinity of peritubular blood vessels identified by red cells. Therefore, we isolated human CLCNKA positive tubular epithelial cells (CLCNKA+ hTECs; **Figure** **2**A), human AQP2 positive (AQP2+) collecting duct cells (AQP2+ hCDCs; Figure 2A), hRIFs (Figure 2A), and hRPCECs (Figure 2A), and further investigated the osteogenic‐like potential of these cells when subjected to osteogenic medium. Both ARS staining (Figure 2B,C) and ALP activity assay (Figure 2D) indicated that hRIFs exhibited the highest osteogenic‐like potential among these cells. Additionally, immunofluorescence costaining showed that fibroblasts, identified by vimentin staining, were partially colocalized with the upregulated osteogenic markers (RUNX2; OCN) within RP tissues (Figure 2E–H). These findings suggested that osteogenic‐like hRIFs might contribute to the CaP deposition observed in the renal interstitium of RP tissues.

### 2.3 OMD Promoted the Osteogenic‐Like Differentiation of hRIFs

**Figure 3:**
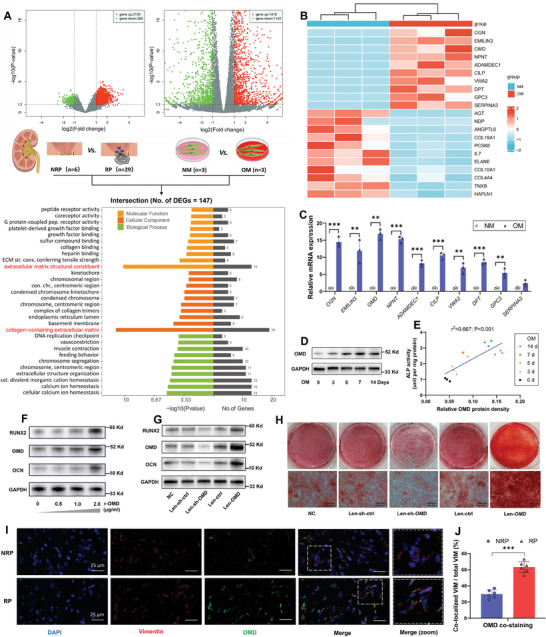
OMD promoted the osteogenic‐like differentiation of hRIFs. A) Transcriptome RNA‐sequencing screened differentially expressed genes (DEGs) in RP. Randall's plaque (RP) tissues and osteogenic‐induced hRIFs, and GO analysis for the intersection of DEGs were performed. NRP, normal renal papillae; NM, normal medium; OM, osteogenic medium. B,C) From DEGs of osteogenic‐induced hRIFs, genes enriched in extracellular matrix structure constituents and components containing collagen‐containing extracellular matrix were obtained. A heatmap was used to visualize the top ten upregulated and downregulated genes, and qRT‐PCR determined the top ten upregulated genes; *n* = 3. D) WB determined OMD in hRIFs cultured with OM for 0, 3, 5, 7, and 14 days. E) The correlation of OMD protein expression and ALP activity in hRIFs cultured with OM for 0, 3, 5, 7, and 14 days. F) hRIFs were cocultured with recombinant human OMD protein (r‐OMD) for 7 days, and WB determined osteogenic markers (OCN, RUNX2) and OMD. G) OMD was either overexpressed or silenced in hRIFs using recombinant lentivirus (Len), and WB determined osteogenic markers and OMD after osteogenic induction for 7 days; *n* = 3. H) Alizarin red staining for hRIFs with overexpressed or silenced *OMD* after osteogenic induction for 14 days; *n* = 3. I,J) Immunofluorescence costaining for VIM and RUNX2 in NRP (*n* = 6) and RP (*n* = 6) tissues, and the percentage of the colocalized VIM to the total VIM were calculated.

To explore the potential factors regulating osteogenic‐like differentiation of hRIFs to participate in RP formation, we obtained transcriptome RNA‐seq data on RP (*n* = 29) and NRP (normal renal papillae, *n* = 6) tissues from GEO (GSE73680) and performed transcriptome RNA‐seq of hRIFs cultured in osteogenic (*n* = 3) or normal (*n* = 3) medium. Compared with NRP tissues, RP tissues showed upregulation of 2130 mRNAs and downregulation of 266 mRNAs (**Figure** **3**A). Compared with hRIFs cultured in normal medium, hRIFs cultured in osteogenic medium showed upregulation of 1416 mRNAs and downregulation of 1142 mRNAs (Figure 3A). There were 147 mRNAs in the intersection of these differentially expressed genes (DEGs), and Gene Ontology (GO) analysis based on these 147 mRNAs revealed that the most statistically significant molecular functional module was extracellular matrix structure constituent, and the most statistically significant cellular component module was collagen‐containing extracellular matrix (Figure 3A). It indicated that genes related to the extracellular matrix might be involved in the osteogenic‐like differentiation of hRIFs and the formation of Randall's plaques. To further explore the extracellular matrix‐related factors that may modulate the osteogenic differentiation of hRIFs, we extracted genes enriched in extracellular matrix structure and collagen‐containing extracellular matrix components from DEGs in hRIFs with osteogenic induction, and the top ten upregulated and top ten downregulated mRNAs were displayed in a heatmap (Figure 3B).

To explore the potential factors promoting the osteogenic‐like differentiation of hRIFs, we further focused on those top ten upregulated mRNAs. Quantitative real‐time polymerase chain reaction (qRT‐PCR) verified that *OGN*, *EMILIN3*, *OMD*, *NPNT*, *ADAMDEC1*, *CILP*, *VWA2*, *DPT*, and *GPC3* mRNA expressions were upregulated (Figure 3C), among which *OMD* mRNA expression exhibited the highest fold change (FC = 16.7). Therefore, we interrogated the role of *OMD* in osteogenic‐like differentiation of hRIFs. The expression of OMD protein increased in a time‐dependent manner following osteogenic induction (Figure 3D; Figure S1A, Supporting Information), and the relative OMD protein density was positively correlated to ALP activity (Figure 3E; Figure S1B, Supporting Information) of hRIFs during the osteogenic induction. Additionally, recombinant human OMD protein (r‐OMD) enhanced the osteogenic‐like phenotype of hRIFs in a concentration‐dependent manner (0.5–2.0 µg mL^−1^), as elucidated by the upregulated expression of osteogenic proteins (Figure 3F; Figure S1C–E, Supporting Information), the increased ALP activity (Figure S1F, Supporting Information), and calcium depositions (Figure S1G,H, Supporting Information) in cell layers secondary to r‐OMD coculture. Moreover, we utilized constructed lentivirus to either overexpress or silence *OMD* expression in hRIFs (Figure S1I, Supporting Information), and confirmed that OMD played a promotive role in the osteogenic‐like phenotype of hRIFs (Figure 3G,H; Figure S1J–L, Supporting Information). Meanwhile, we compared OMD protein expression in RP and NRP tissues, and found the upregulated OMD in hRIFs (Figure 3I,J) within RP tissues, but not in renal tubular epithelial cells marked by E‐cadherin (Figure S2A; Figure S2C, Supporting Information) or peritubular capillary endothelial cells marked by CD34 (Figure S2B; Figure S2D, Supporting Information). These results indicated an osteogenic‐like phenotype of hRIFs with upregulated OMD as a pathological feature of RP tissues.

### 2.4 OMD Involved in the Osteogenic‐Like Microenvironment of RP Tissues

**Figure 4:**
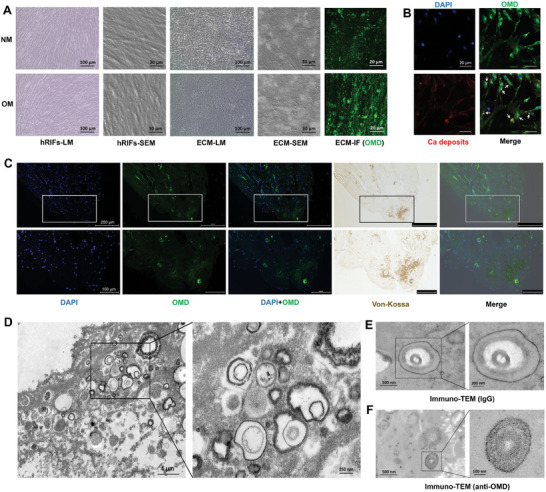
OMD contributed to the osteogenic‐like microenvironment of RP tissues. A) After 14 days of culture in normal medium (NM) or osteogenic medium (OM), light microscopy (LM), and scanning electron microscopy (SEM) determined the characteristics of hRIFs and extracellular matrix (ECM) extracted from hRIFs, and immunofluorescence (IF) determined OMD in ECM; *n* = 4. B) IF costaining for OMD and calcium deposits in hRIFs induced with OM for 14 days; *n* = 3. C) Colocalization of OMD stained by IF and calcium deposits stained by Von‐Kossa within RP tissues; *n* = 3. D) Transmission electron microscopy (TEM) revealed calcium vesicles in RP tissues, presenting as spheres with alternating light and dark rings; *n* = 3. E,F) Immuno‐TEM detected OMD in those vesicles; *n* = 3. IgG was utilized as the negative control for anti‐OMD, and the black gold particles indicated the positive expression of OMD.

As described in The Human Protein Atlas (https://www.proteinatlas.org), OMD belongs to the family of small leucine‐rich proteoglycans consisting of leucine‐rich repeats (Figure S3A, Supporting Information), and is predicted to be a secreted extracellular matrix (ECM) protein. We further prepared the ECM of hRIFs and confirmed its morphology through SEM (**Figure** **4**A). Immunofluorescence revealed a significant upregulation of OMD in the ECM of hRIFs following osteogenic induction (Figure 4A; Figure S3B, Supporting Information). Additionally, SEM coupled with EDS suggested widespread calcium salt deposits in ECM (Figure S3C, Supporting Information), which echoed that OMD was colocalized with calcium deposition in cell layers of hRIFs (Figure 4B). Consistent with our findings in vitro, costaining of immunofluorescence and Von‐Kossa demonstrated high colocalization of OMD expression and calcium depositions (Figure 4C) within RP tissues. Moreover, calcium vesicles, appearing as spheres with alternating light and dark rings (Figure 4D), were detected in RP tissues by transmission electron microscopy (TEM). OMD was further detected in those vesicles using immunoelectron microscopy (Figure 4E,F). The above results indicated that OMD was involved in the osteogenic‐like microenvironment of renal interstitium to participate in RP formation.

### 2.5 OMD Enhanced BMP2 Protein Stability via Inhibiting E3 Ligase NEDD4‐Mediated Ubiquitination of BMP2

**Figure 5:**
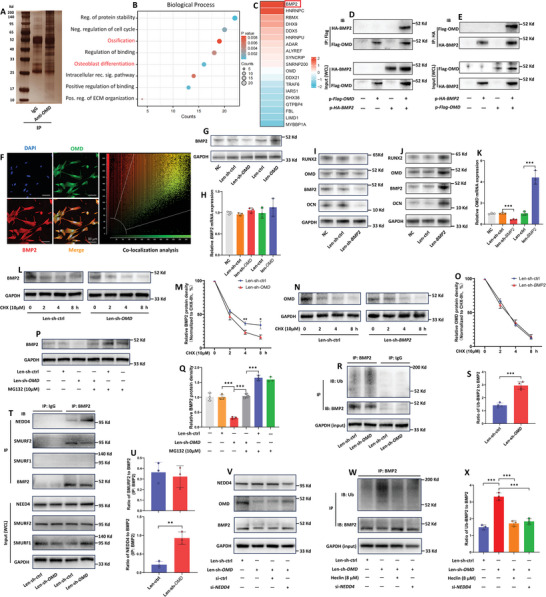
OMD interacted with BMP2 and inhibited the ubiquitination of BMP2. A) Proteins in anti‐OMD or IgG (negative control) immune‐precipitates from the whole cell lysate of hRIFs were resolved by 12% SDS‐PAGE and visualized by silver staining. B) Proteins in immune‐precipitates were analyzed with proteomics by mass spectrometry, and biological process of GO analysis was performed on 468 proteins with a fold change of more than 8 in the anti‐OMD group compared to the IgG group. C) A total of 19 proteins related to ossification or/and osteoblast differentiation was listed in the heatmap, showing BMP2 with the highest abundance in anti‐OMD immune‐precipitates. D,E) hRIFs were transfected with constructed plasmids overexpressing *BMP2* with an HA tag (p‐HA‐*BMP2*) or/and plasmids overexpressing *OMD* with a Flag tag (p‐Flag‐*OMD*), and WB was then employed to analyze the expression of Flag‐*OMD* and HA‐*BMP2* in immune‐precipitates; *n* = 3. F) Fluorescence‐labeled OMD (green) and BMP2 (red) in hRIFs; *n* = 3, with a mean colocalization ratio of 75.6%. G,H) Protein and mRNA expressions of BMP2 were determined in hRIFs with either overexpression or silence of *OMD*, seven days after osteogenic induction; *n* = 3. I‐J) Protein expressions of BMP2, OCN, RUNX2, and OMD were determined in hRIFs with either overexpression or silence of *BMP2*, seven days after osteogenic induction; *n* = 3. K) *OMD* mRNA expressions were determined in hRIFs with either overexpressed or silenced *BMP2*, seven days after osteogenic induction; *n* = 3. L,M) hRIFs transfected with Len‐sh‐ctrl or Len‐sh‐*OMD* were pretreated with CHX (10 µm), and BMP2 was determined by WB at indicated time points; *n* = 3. N,O) hRIFs transfected with Len‐sh‐ctrl or Len‐sh‐*BMP2* were pretreated with CHX (10 µm), and OMD was determined by WB at indicated time points; *n* = 3. P,Q) hRIFs transfected with Len‐sh‐ctrl or Len‐sh‐*OMD* were pretreated with or without the proteasome inhibitor MG‐132 (10 µm) for 8 h, and BMP2 was determined by WB; *n* = 3. R,S) hRIFs transfected with Len‐sh‐ctrl or Len‐sh‐*OMD* were pretreated with MG‐132 for 8 h, and the immune‐precipitates of anti‐BMP2 or IgG (negative control) were incubated with anti‐ubiquitin; *n* = 3. The ubiquitin level of BMP2 was normalized to the total of BMP2 in immune‐precipitates. T,U) hRIFs were transfected with Len‐sh‐ctrl or Len‐sh‐*OMD*, and E3 ubiquitin ligases (SMURF1; SMURF2; NEDD4) were determined in the whole cell lysis (WCL) or IgG/anti‐BMP2 immune‐precipitates; *n* = 3. E3 ubiquitin ligases were normalized to BMP2 in anti‐BMP2 immune‐precipitates. V) hRIFs were cotransfected with Len‐sh‐*OMD* and si‐*NEDD4*; BMP2, OMD, and NEED4 were determined; *n* = 3. W,X) hRIFs were treated with NEDD4 inhibitor (Heclin; 8 µm) or si‐*NEDD4*, and the ubiquitin level of BMP2 was determined in hRIFs transfected with Len‐sh‐ctrl or Len‐sh‐*OMD*; *n* = 3.

To explore the underlying mechanism how OMD enhances the osteogenic‐phenotype of hRIFs, we utilized co‐immunoprecipitation (Co‐IP) combined with proteomics by mass spectrometry to explore the potential proteins binding to OMD (**Figure** **5**A). Compared to IgG immune‐precipitates (Table S8, Supporting Information), there were 468 proteins with a fold change of more than 8 in anti‐OMD immune‐precipitates (Table S9, Supporting Information). GO analysis of biological process based on these proteins revealed that the top five statistically significant modules included ossification and osteoblast differentiation (Figure 5B), both of which were closely associated with the promotive role of OMD in osteogenic‐like differentiation of hRIFs. Among proteins enriched in the above two functional modules, BMP2 exhibited the highest abundance in anti‐OMD immune‐precipitates (Figure 5C), and we further confirmed the direct binding of OMD to BMP2 using Co‐IP followed by WB (Figure 5D,E). Additionally, immunofluorescence staining showed that OMD partially colocalized with BMP2 in hRIFs under osteogenic conditions, primarily in the cytoplasm (Figure 5F).

Considering the binding of BMP2 to OMD, we investigated whether BMP2 and OMD mutually alter their protein expression. OMD promoted BMP2 protein expression (Figure 5G; Figure S3D, Supporting Information), and BMP2 also enhanced OMD protein expression (Figure 5I,J; Figure S3E–H, Supporting Information). Additionally, BMP2 enhanced the expression of osteogenic markers (RUNX2; OCN; Figure 5I,J; Figure S3E–H, Supporting Information), indicating an important role of BMP2 in osteogenic‐like differentiation of hRIFs. Meanwhile, we also determined the mRNA expression of *BMP2* and *OMD*, and qRT‐PCR results showed that BMP2 upregulated *OMD* mRNA expression (Figure 5K), while *BMP2* mRNA expression remained unchanged under conditions of *OMD* overexpression or silence (Figure 5H). These results suggested that OMD might regulate *BMP2* expression at the post‐transcriptional level, and BMP2 might regulate *OMD* expression at least transcriptionally. It prompted us to explore whether OMD and BMP2 mutually mediate their degradations, since BMP2 interacted with OMD. Utilizing the protein synthesis inhibitor cycloheximide (CHX), we found that *OMD* silence markedly shortened the half‐life of BMP2 (Figure 5L,M), while there was no effect of *BMP2* silence on the half‐life of OMD (Figure 5N,O). Considering that the ubiquitin‐proteasome pathway is the primary route for protein degradation of short‐lived proteins,^[^ ^26^ ^]^ we speculated that OMD might inhibit the degradation of BMP2 via disrupting the ubiquitin‐proteasome system. Subsequently, hRIFs were transfected with Len‐sh‐*OMD* and then treated with the proteasome inhibitor MG‐132, which markedly restored the protein level of BMP2 suppressed by *OMD* silence (Figure 5P,Q). Furthermore, *OMD* silence dramatically increased the ubiquitination level of BMP2 (Figure 5R,S).

Given that E3 ubiquitin ligases target substrate proteins for ubiquitination in eukaryotes,^[^ ^27^ ^]^ and E3 ubiquitin ligase SMURF1,^[^ ^28^ ^]^ SMURF2,^[^ ^29^ ^]^ and NEDD4^[^ ^30^ ^]^ were reported to regulate both BMP signaling pathway and ossification‐related processes, we explored whether these E3 ligases are involved in the OMD‐inhibited ubiquitination of BMP2. Initially, we investigated whether OMD affects the protein expression of SMURF1, SMURF2, and NEDD4. However, silencing OMD in hRIFs did not affect their expression (Figure 5T; Figure S3I, Supporting Information). This prompted us to investigate whether OMD influences the interaction of BMP2 and these E3 ligases, given OMD's ability to bind BMP2. Co‐IP showed that BMP2 bound to E3 ligase SMURF2 and NEDD4, but not SMURF1 (Figure 5T). Notably, *OMD* silence significantly enhanced the binding of BMP2 to NEDD4, but not to SMURF2 (Figure 5T,U). Additionally, si‐*NEDD4* restored the BMP2 protein expression decreased by *OMD* silence (Figure 5V; Figure S3J, Supporting Information), and using NEDD4 inhibitor Heclin produced consistent results (Figure S3K, Supporting Information). Furthermore, both si‐*NEDD4* and Heclin largely reduced the ubiquitination of BMP2 that was promoted by *OMD* silence (Figure 5W,X). These findings indicated that OMD enhanced BMP2 protein stability via inhibiting E3 ligase NEDD4‐mediated ubiquitination of BMP2.

### 2.6 OMD/BMP2/BMPR1A/RUNX2/OMD Developed a Positive Feedback Loop to Promote the Osteogenic‐Like Differentiation of hRIFs

**Figure 6:**
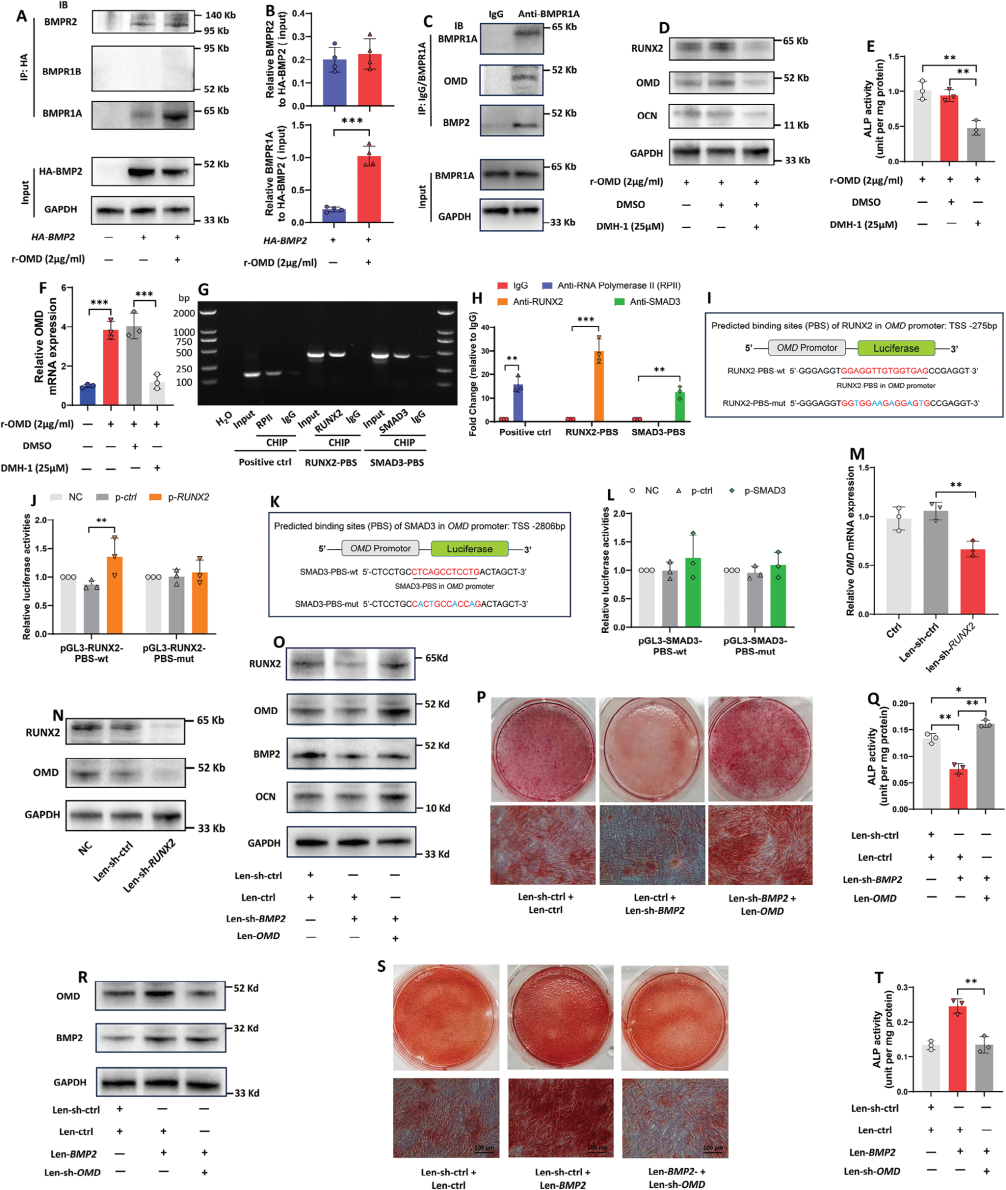
OMD/BMP2/BMPR1A/RUNX2/OMD axis promoted the osteogenic‐like differentiation of hRIFs. A,B) hRIFs were transfected with plasmids carrying BMP2 with an HA tag (p‐HA‐BMP2) and induced with osteogenic medium (OM) for 7 days. Anti‐HA immunoprecipitation (IP) was performed to evaluate the role of recombinant human OMD (r‐OMD, 2 µg mL^−1^) in the binding of BMP2 to its membrane receptors (BMPR1A, BMPR1B, BMPR2). The density of BMPR1A and BMPR2 in immune‐precipitates were normalized to the density of HA‐BMP2; *n* = 4. C) BMP2 and OMD were determined in the anti‐BMPR1A immunoprecipitation; *n* = 3. D–F) hRIFs were cocultured with r‐OMD (2 µg mL^−1^) and DMH‐1 (25 µm), a selective inhibitor of BMPR1, in OM for 7 days, and the protein expression (OCN; OMD; RUNX2) and alkaline phosphatase (ALP) activity were determined; *n* = 3. F) hRIFs were cocultured with or without r‐OMD or/and DMH‐1 in OM for 7 days, and qRT‐PCR determined *OMD* mRNA expression; *n* = 3. G) Agarose gel electrophoresis showed the PCR products of input and chromatin immunoprecipitation (ChIP). Positive control was performed by amplifying the *GAPDH* promoter in RNA polymerase II (RPII)‐ChIP; IgG as a negative control. RUNX2‐PBS/SMAD3‐PBS, predicted binding sites of RUNX2/SMAD3 in *OMD* promoter. H) ChIP products were analyzed by q‐PCR, expressed as the fold change to the IgG group; *n* = 3. I,J) A schematic diagram illustrating the pGL3‐basic luciferase reporter vector carrying the sequence of RUNX2‐PBS (RUNX2‐PBS‐wt) or the mutant sequence (RUNX2‐PBS‐mut). hRIFs were cotransfected with either p‐ctrl or p‐*RUNX2* and either pGL3‐RUNX2‐PBS‐wt or pGL3‐RUNX2‐PBS‐mut, and the relative luciferase activity (firefly/renilla) was determined; *n* = 3. TSS ‐, upstream of transcriptional start. K,L) hRIFs were cotransfected with either p‐ctrl or p‐*SMAD3* and either pGL3‐SMAD3‐PBS‐wt or pGL3‐SMAD3‐PBS‐mut, and the relative luciferase activity (firefly/renilla) was determined; *n* = 3. M,N) OMD and RUNX2 were determined in hRIFs transfected with Len‐sh‐*RUNX2* after osteogenic induction for 7 days; *n* = 3. O–Q) hRIFs were cotransfected with Len‐sh‐BMP2 and either Len‐ctrl or Len‐*OMD*. Protein expressions and ALP activity were determined after osteogenic induction for 7 days; *n* = 3, and alizarin red staining (ARS) was performed after osteogenic induction for 14 days; *n* = 3. R–T) hRIFs were cotransfected with Len‐*BMP2* and either Len‐ctrl or Len‐sh‐*OMD*. Protein expression analysis, ARS, and ALP activity assay were performed in the indicated time points after osteogenic induction; *n* = 3.

Our above results suggested that OMD and BMP2 had a synergistic effect on osteogenic‐like differentiation of hRIFs, and it has been well established that BMP2, interacting with its cell membrane receptors, plays a vital role in the BMP signaling pathway to regulate osteogenic differentiation.^[^ ^31^ ^]^ Considering that both OMD and BMP2 are ECM‐related proteins, and our study also revealed the strong binding of BMP2 and OMD in the ECM from osteogenic‐induced hRIFs (Figure S4A, Supporting Information), we further investigated the effect of OMD on the interaction of BMP2 and its receptors. Co‐IP showed that BMP2 bound to BMPR1A and BMPR2, but not to BMPR1B in hRIFs under osteogenic conditions (**Figure** **6**A). Notably, hRIFs cocultured with r‐OMD significantly enhanced the interaction of BMP2 with BMPR1A, but had no significant effect on the interaction with BMPR2 (Figure 6A,B). Moreover, both BMP2 and OMD were detected in the anti‐BMPR1A immunoprecipitation (Figure 6C). These results suggested that OMD interacted BMP2 to facilitate its binding to BMPR1A. Utilizing DMH‐1 which specifically targets the intracellular kinase domain of BMPR1 to block the BMP signaling pathway, we revealed that DMH‐1 markedly abolished the effect of r‐OMD on osteogenic‐like differentiation of hRIFs (Figure 6D,E; Figure S4B–D, Supporting Information). Of note, r‐OMD coculture promoted OMD protein (Figure 3F) and mRNA (Figure 6F) expression, which was also diminished by DMH‐1 (Figure 6D,F), indicating *OMD* as the potential target gene regulated by BMP2/BMPR1 signaling.

As predicted by TF‐motifs^[^ ^32^ ^]^ based on JASPAR dataset^[^ ^33^ ^]^ and HOCOMOCO dataset^[^ ^34^ ^]^ using FIMO software,^[^ ^35^ ^]^ there were 127 transcription factors potentially binding to *OMD* promoter (Table S10, Supporting Information), among which RUNX2^[^ ^36^ ^]^ and SMAD3^[^ ^37^, ^38^ ^]^ have been reported as downstream targets of BMP2/BMPR1A signaling pathway. Therefore, we performed chromatin immunoprecipitation (ChIP) and q‐PCR to determine whether RUNX2 and SMAD3 bind to *OMD* promoter. The ChIP system was validated by including anti‐RNA Polymerase II as the positive control (Figure 6G,H), and we revealed that RUNX2 and SMAD3 could bind to *OMD* promoter as predicted (Figure 6G,H). The dual‐luciferase reporter assay further demonstrated that RUNX2 could bind to the predicted region of *OMD* promoter to upregulate downstream gene encoding firefly luciferase (Figure 6I,J), while SMAD3 had no such effect (Figure 6K,L). Consistently, silence of *RUNX2* (Figure S4E,F, Supporting Information) significantly suppressed *OMD* mRNA expression (Figure 6M), leading to the downregulation of OMD protein in hRIFs (Figure 6N; Figure S4F, Supporting Information).

Our study revealed that OMD promoted BMP2 protein and enhanced the binding of BMP2 to its receptor BMPR1A, resulting in the upregulation of RUNX2, which further promoted *OMD* mRNA expression via binding to *OMD* promoter. These results indicated an OMD/BMP2/BMPR1A/RUNX2/OMD axis that promoted the osteogenic‐like differentiation of hRIFs. To further verify whether the axis develops a positive feedback loop, hRIFs were cotransfected with Len‐sh‐*BMP2* and Len‐*OMD*, or cotransfected with Len‐*BMP2* and Len‐sh‐*OMD*. Overexpression of *OMD* significantly recovered the osteogenic‐like phenotype of hRIFs suppressed by the silence of *BMP2* (Figure 6O–Q; Figure S4G–K, Supporting Information). Consistently, silence of *OMD* significantly recovered the osteogenic‐like phenotype enhanced by overexpression of *BMP2* (Figure 6R–T; Figure S4L–N, Supporting Information). Combined with the observation that r‐OMD promoted the protein expression of OMD, BMP2, and RUNX2 (Figure 3F), our findings suggested that the OMD/BMP2/BMPR1A/RUNX2/OMD axis developed a positive feedback loop to promote the osteogenic‐like differentiation of hRIFs.

### 2.7 OMD Promoted BMP2 as well as Osteogenic‐Like Phenotype of hRIFs In Vivo

**Figure 7:**
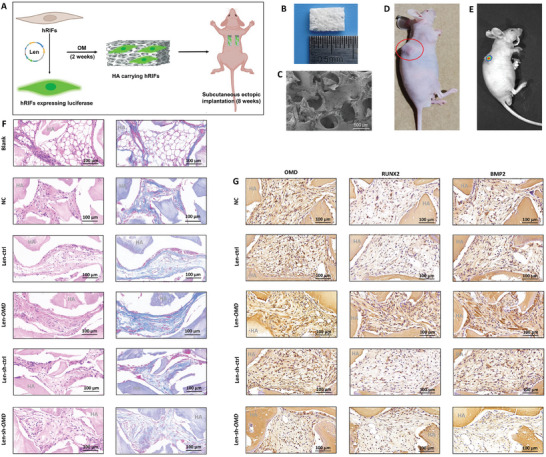
OMD promoted BMP2 and osteogenic‐like phenotype of hRIFs in vivo. A) A schematic diagram illustrating that hRIFs transfected with recombinant lentivirus (Len) encoding luciferase were introduced to monitor their survival 8 weeks after subcutaneous implantation in nude mice. OM, osteogenic medium. B) Hydroxyapatite (HA) scaffolds. C) The porous structure of HA scaffolds visualized by scanning electron microscopy. E) Fluorescence was detected in subcutaneous implants 8 weeks post‐implantation of luciferase‐expressing hRIFs following injection of d‐Luciferin; *n* = 3. F) Serial sections from the following groups were subjected to HE and Masson staining: the blank group (grafts without cells); the normal control (NC) group (grafts with hRIFs); the Len‐ctrl group (grafts with hRIFs transfected with Len‐ctrl); the Len‐*OMD* group (grafts with hRIFs transfected with Len‐*OMD*); the Len‐sh‐ctrl group (grafts with hRIFs transfected with Len‐sh‐ctrl); and the Len‐sh‐*OMD* group (grafts with hRIFs transfected with Len‐sh‐*OMD*); *n* = 6. G) Immunohistochemical staining for OMD, RUNX2, and BMP2 was performed on serial sections from the NC group, the Len‐ctrl group, the Len‐*OMD* group, the Len‐sh‐ctrl group, and the Len‐sh‐*OMD* group; *n* = 6.

To determine the role of OMD in the osteogenic‐like phenotype of hRIFs in vivo, we subcutaneously implanted HA scaffolds carrying osteogenic‐induced hRIFs in nude mice (**Figure** **7**A–D). Initially, hRIFs transfected with luciferase (Figure S5A, Supporting Information) were introduced to monitor their survival post‐implantation (Figure 7A). Fluorescence imaging of the dorsal implants in nude mice revealed that hRIFs persisted for 8 weeks following subcutaneous implantation (Figure 7E). This observation was corroborated by our findings that the blank group exhibited void bubble‐like formations, contrasting with the organized collagen fibers present in implants containing hRIFs after implantation for 8 weeks (Figure 7F). Furthermore, histological staining with HE or Masson's trichrome revealed that *OMD* overexpression notably augmented collagen fibers, whereas *OMD* silence substantially reduced collagen fibers within implants (Figure 7F; Figure S5B, Supporting Information). Additionally, IHC staining demonstrated that *OMD* overexpression upregulated and BMP2 and RUNX2 (Figure 7G; Figure S5C, Supporting Information). Conversely, *OMD* silence significantly inhibited BMP2 but not RUNX2 (Figure 7G; Figure S5C, Supporting Information). These findings indicated that OMD promoted the osteogenic‐like phenotype of hRIFs in vivo. Notably, the transplanted hRIFs survived long‐term under the skin of nude mice, a nonosteogenic environment, but BMP2 remained upregulated in hRIFs with OMD overexpression, which mirrored the positive feedback loop between OMD and BMP2 identified in vitro.

### 2.8 *Col1a2‐CreERT^tg/+^; Omd^flox/flox^* Mice Were Protected from CaOx Nephrocalcinosis Induced by a High Hydroxyproline Diet and Subcutaneous Injection of VD_2_

**Figure 8:**
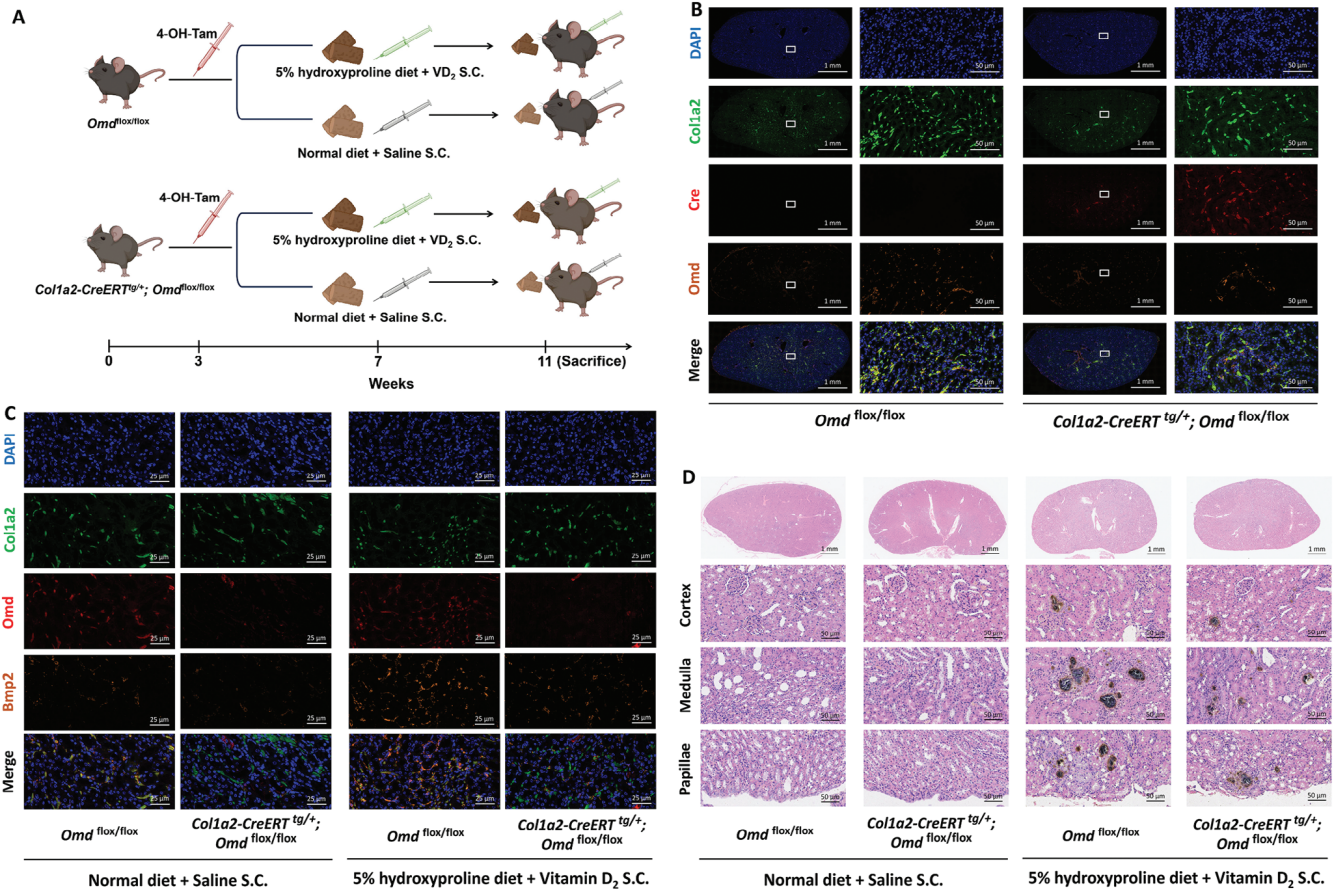
CaOx nephrocalcinosis was attenuated in *Col1a2‐CreERT^tg/+^*; *Omd^flox/flox^* mice treated with a high hydroxyproline diet and subcutaneous injection of VD_2._ A) A schematic diagram illustrating CaOx nephrocalcinosis induced in *Omd^flox/flox^* mice and *Col1a2‐CreERT^tg/+^*; *Omd^flox/flox^* mice. All mice were injected with 4‐OH tamoxifen (4‐OH‐Tam) intraperitoneally from 5 to 11 weeks of age. From 7 to 11 weeks of age, mice received a 5% hydroxyproline diet and subcutaneous injection (S.C.) of VD_2_ to induce CaOx nephrocalcinosis, and the control group received a normal diet and S.C. of saline. B) Before inducing CaOx nephrocalcinosis, immunofluorescence costaining was performed to assess the efficiency of conditional *Omd* knockout in Col1a2‐marked renal interstitial fibroblasts following a three‐week intraperitoneal injection of 4‐OH‐Tam. *Omd^flox/flox^* mice, *n* = 3; *Col1a2‐CreERT^tg/+^*; *Omd^flox/flox^* mice, *n* = 3. C,D) *Omd^flox/flox^* mice and *Col1a2‐CreERT^tg/+^*; *Omd^flox/flox^* mice either underwent induction of CaOx nephrocalcinosis or were assigned to a control group. Immunofluorescence costaining of Omd and Bmp2 was performed in Col1a2‐marked renal interstitial fibroblasts. Von‐Kossa staining was performed to assess calcium deposits in renal cortex, medulla, and papillae. Normal diet + Saline S.C. group (*Omd^flox/flox^* mice, *n* = 9; *Col1a2‐CreERT^tg/+^*; *Omd^flox/flox^* mice, *n* = 8); 5% hydroxyproline diet + Vitamin D_2_ S.C. group (*Omd^flox/flox^* mice, *n* = 9; *Col1a2‐CreERT^tg/+^*; *Omd^flox/flox^* mice, *n* = 7).

We generated *Col1a2‐CreERT^tg/+^*; *Omd^flox/flox^* mice to conditionally knock out *Omd* in renal interstitial fibroblasts (Figure S5D–F, Supporting Information), and further determined the role of *Omd* in CaOx nephrocalcinosis induced by high hydroxyproline diet and subcutaneous VD_2_ injection (**Figure** **8**A). Before CaOx nephrocalcinosis induction, immunofluorescence confirmed the efficiency of *Omd* knockout in *Col1a2‐CreERT^tg/+^*; *Omd^flox/flox^* mice following a three‐week intraperitoneal injection of 4‐OH‐Tam (Figure 8B). Subsequently, mice were subjected to CaOx nephrocalcinosis induction or a normal diet with subcutaneous saline injection as a control for four weeks. In *Omd^flox/flox^* mice undergoing CaOx nephrocalcinosis induction, Omd, Bmp2, and Runx2 were upregulated in renal interstitial fibroblasts, and *Omd* knockout markedly suppressed the upregulated Bmp2 and Runx2 (Figure 8C; Figures S5G and S6, Supporting Information). Of note, in those mice subjected to a normal diet and subcutaneous saline injection, Bmp2 expression was also lower in *Col1a2‐CreERT^tg/+^*; *Omd^flox/flox^* mice compared to *Omd^flox/flox^* mice (Figure 8C; Figure S5G, Supporting Information). Moreover, CaOx nephrocalcinosis was significantly attenuated in *Col1a2‐CreERT^tg/+^*; *Omd^flox/flox^* mice compared to *Omd^flox/flox^* mice, especially in the renal medulla and renal papillae (Figure 8D; Figure S5H, Supporting Information).

### 2.9 OMD Was Upregulated and Correlated to the Increased Osteogenic Markers in RP Tissues

**Figure 9:**
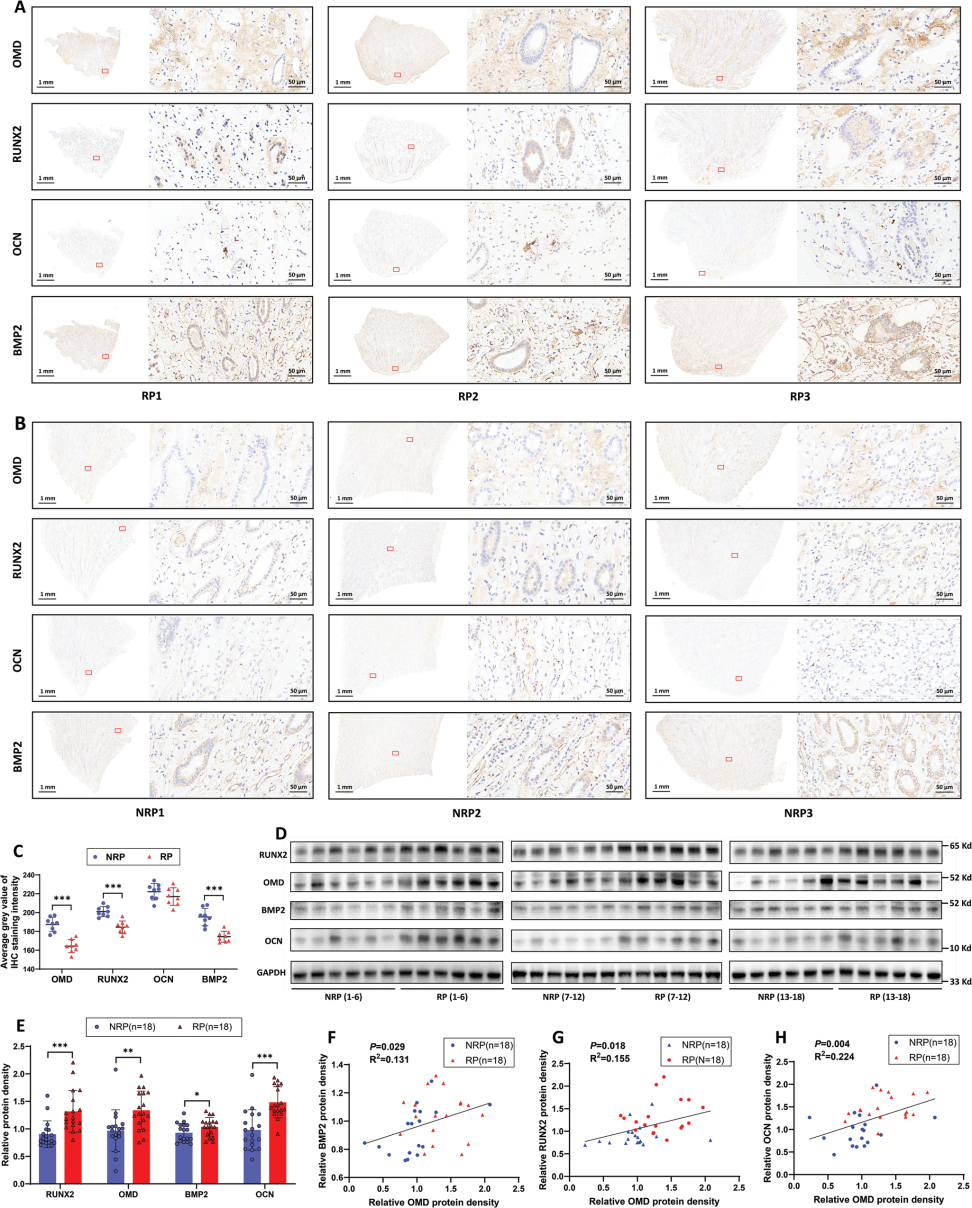
Upregulated OMD was correlated to osteogenic markers in Randall's plaque (RP) tissues. A–C) Immunohistochemistry (IHC) staining of OMD, RUNX2, OCN, and BMP2 in RP tissues and normal renal papillae (NRP). IHC staining intensity was quantified by average gray value using ImageJ; *n* = 8 for each group. The average gray value ranged from 0 to 254, where a black, dark‐stained area was assigned a gray value of 0, and a white, unstained area was assigned a gray value of 254. This setup resulted in an inverse correlation between the average gray value and staining intensity. D,E) OMD, RUNX2, OCN, and BMP2 were determined by WB in NRP (*n* = 18) and RP (*n* = 18) tissues, and WB bands were quantified by ImageJ and normalized to GAPDH. F–H) The correlation of relative OMD protein expression to BMP2, RUNX2, and OCN expressions, determined by WB in NRP (*n* = 18) and RP (*n* = 18) tissues.

To further evaluate the potential roles of OMD, BMP2, and other osteogenic‐related markers (RUNX2, OCN) in RP formation, IHC staining was performed on RP and NRP tissues (**Figure** **9**A,B). Utilizing semiquantitative analysis of IHC, we found that OMD, RUNX2, and BMP2 proteins were significantly elevated in RP tissues, while OCN showed no significant difference (Figure 9C). Additionally, WB demonstrated an elevated protein expression of OMD, BMP2, RUNX2, and OCN in RP tissues compared to NRP tissues (Figure 9D,E), and the relative expression level of OMD protein was positively correlated to BMP2, RUNX2, and OCN (Figure 9F–H).

**Figure 10:**
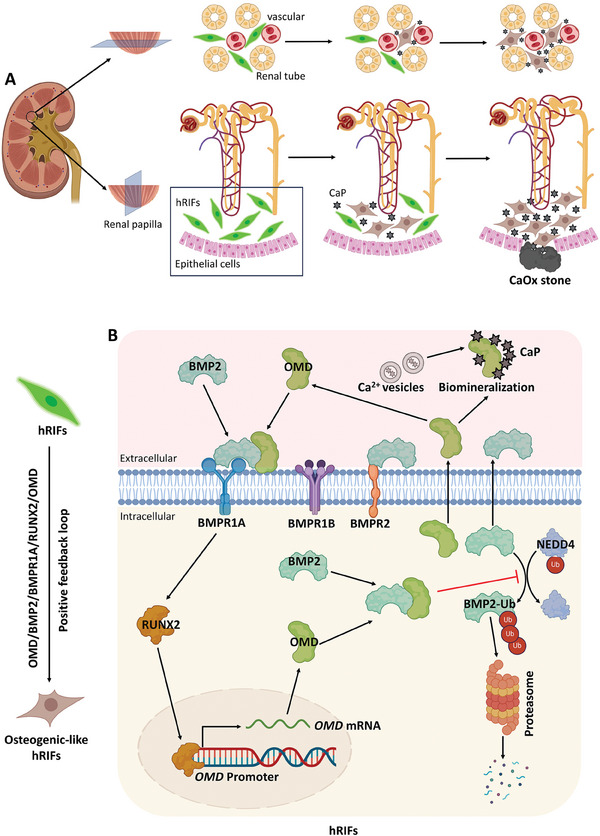
A schematic diagram illustrating that osteogenic microenvironment of renal interstitium induced by OMD contributed to Randall's plaque (RP) formation. A) hRIFs adopted an osteogenic‐like phenotype, creating an osteogenic microenvironment in the renal interstitium where calcium phosphate (CaP) crystals deposited. These crystals grown outward and eventually breached the renal papillary surface, serving as an attachment for CaOx crystals to develop CaOx stones. B) OMD bound to BMP2 and inhibited NEDD4‐mediated ubiquitination of BMP2 protein, thereby preventing its degradation through the proteasomal pathway. Simultaneously, OMD enhanced the BMP2's binding to its membrane receptor BMPR1A, leading to upregulation of the osteogenic transcription factor RUNX2, a downstream factor of BMP2/BMPR1A signaling. RUNX2 further promoted the transcription of *OMD* via binding to the *OMD* promoter. Taken together, there was a positive feedback loop of OMD/BMP2/BMPR1A/RUNX2/OMD promoting the osteogenic‐like differentiation of hRIFs, through which OMD induced the osteogenic microenvironment of renal interstitium to participate in RP formation. This illustration was created with BioRender.com.

Taken together, these results suggested a positive feedback loop of OMD/BMP2/BMPR1A/RUNX2/OMD promoting the osteogenic‐like differentiation of hRIFs, through which OMD induced the osteogenic‐like microenvironment of renal interstitium to participate in RP formation (**Figure** **10**A,B).

## 3 Discussion

This study reinforced the evidence that RPs served as the nidus for some CaOx stones.^[^ ^39^, ^40^ ^]^ Utilizing the costaining of osteogenic markers and VIM, hRIFs were highlighted as the dominant osteogenic‐like cells in RP tissues. Further analysis revealed that OMD promoted the osteogenic‐like differentiation of hRIFs both in vitro and in vivo. Upregulated OMD was colocalized with calcium deposits in RP tissues, and mice with *Omd* knockout in renal fibroblasts attenuated CaOx nephrocalcinosis. Our study for the first time revealed that OMD enhanced the osteogenic‐like phenotype of hRIFs and induced the osteogenic‐like microenvironment of renal interstitium to participate in RP formation.

OMD was first reported by Wendel et al. who utilized guanidine hydrochloride‐containing EDTA to extract it from bovine bone.^[^ ^41^ ^]^ A few studies revealed OMD as a promotive regulator in biomineralization. Zhu et al. reported that *OMD* promoted osteogenic differentiation of human mesenchymal progenitor cells.^[^ ^42^ ^]^ Additionally, OMD was found to play a critical role in promoting the osteo/odontoblastic differentiation of human dental pulp stem cells (hDPSCs).^[^ ^43^, ^44^ ^]^ Consistently, we revealed that OMD promoted osteogenic‐like differentiation of hRIFs both in vitro and in vivo. Intriguingly, Wendel et al. also identified that OMD bound to hydroxyapatite,^[^ ^41^ ^]^ which supported our findings that OMD was colocalized with calcium deposits and even enriched in calcium vesicles within RP tissues. Meanwhile, OMD was significantly upregulated in hRIFs within RP tissues, but not in renal tubular epithelial cells, which corroborated our findings that hRIFs had the highest potential of osteogenic‐like differentiation among renal cells.^[^ ^19^ ^]^ Of note, RP formation shared similarities with vascular calcification, in which OMD was also enriched in smooth muscle cells around calcified nodules.^[^ ^45^ ^]^ Given the biomineralization‐associated feature of OMD and its extremely low expression in NRP (https://www.proteinatlas.org/ENSG00000127083‐OMD/tissue), OMD might be a potential molecular marker for ectopic calcification in renal interstitium.

In this work, we highlighted that OMD bound to BMP2, thereby enhancing the stability of intracellular BMP2 and facilitating the interaction of extracellular BMP2 with its receptor BMPR1A. The interaction of OMD and BMP2 was also observed in a previous study where BMP2 formed complexes with the terminal leucine‐rich repeats of OMD.^[^ ^44^ ^]^ In terms of other members of the BMP family, our Co‐IP coupled proteomics by mass spectrometry did not identify the potential members binding to OMD. Given the limited sensitivity of proteomics by mass spectrometry, and that OMD was reported to be positively associated with BMP4/6 in atherosclerotic plaques,^[^ ^46^ ^]^ further investigations were needed to clarify the potential interaction of OMD and other BMPs. Additionally, we revealed that OMD inhibited E3 ligase NEDD4‐mediated the ubiquitination of BMP2, which echoed that Nedd4 suppressed the BMP‐induced osteoblast transdifferentiation of C2C12 cells.^[^ ^30^ ^]^ In addition to the stability of BMP2 enhanced by OMD, we also revealed that OMD facilitated BMP2 to bind to its membrane receptor BMPR1A, which corroborated that OMD and BMP2 had a synergistic effect on osteogenic differentiation of hDPSCs, and both of them were ECM proteins.^[^ ^41^, ^47^ ^]^ Of note, integrins, a family of ECM proteins, were capable of binding to OMD^[^ ^41^, ^48^ ^]^ as well as BMP2,^[^ ^49^ ^]^ and it needs to clarify whether integrins mediate the interaction of OMD, BMP2, and even BMPR1A.

RUNX2, a well‐established target transcription factor of the BMP2/BMPR1A signaling pathway,^[^ ^36^ ^]^ bound to the promoter of *OMD* gene and thus promoted its mRNA expression. This finding well explained that BMP2 promoted mRNA and protein expression of *OMD*, without affecting the degradation of OMD protein. In C2C12 cells, Bmp2 mediated Smad1/4 to upregulate *Omd* mRNA.^[^ ^50^ ^]^ Despite the difference in TFs, these findings indicated that BMP2 might regulate OMD in a conservative manner across different species. Our findings indicated the positive feedback loop of OMD/BMP2/BMPR1A/RUNX2/OMD promoting the osteogenic‐like differentiation of hRIFs. It might induce an osteogenic‐like microenvironment of renal interstitium in renal papillae, partially explaining the continuous accumulation of calcium deposits in RP tissues. Meanwhile, the osteogenic‐like microenvironment might induce renal tubular cells and hRPCECs to adopt an osteogenic‐like phenotype, contributing to the calcium deposits around renal tubes and peritubular blood vessels within RP tissues, as observed in our present and previous studies.^[^ ^23^, ^51^, ^52^ ^]^

RPs consist of calcium phosphate crystals mixed with an organic matrix within which collagen fibers were underscored.^[^ ^53^ ^]^ TEM of RP tissues showed that the fusion of calcified vesicles with calcified collagen produced large calcified deposits surrounded by collagen fibers.^[^ ^10^ ^]^ Chidambaram et al. utilized a decellularized porcine urinary bladder matrix enriched with collagen and a liquid phase amorphous mineral precursor to successfully develop biomimetic RPs,^[^ ^54^ ^]^ in which intrafibrillar mineralization of collagen was akin to calcium deposits of renal interstitium. Intriguingly, OMD was capable of binding to type‐I collagen driven by weak electrostatic forces, through which OMD regulated the growth of collagen fibrils and its structure.^[^ ^55^ ^]^ Further studies are warranted to investigate whether OMD contributes to renal interstitial biomineralization via the synergistic effect of the interaction with collagen fibers and its role in osteogenic‐like differentiation of hRIFs.

In addition to the osteogenic‐like microenvironment, immune response^[^ ^53^ ^]^ and cell apoptosis^[^ ^56^ ^]^ were also implicated in RP formation, and it remains unclear whether OMD is involved in these processes. Bone Omd expression was decreased in mice (*op*/*op*) in lack of functional macrophage colony‐stimulating factor (M‐CSF), and treatment with recombinant M‐CSF attenuated the decreased Omd expression,^[^ ^57^ ^]^ indicating OMD expression was closely associated with immune response. Recently, a spatially anchored transcriptomic atlas of RP tissues revealed immune activation and significant fibroblast infiltration in calcification areas,^[^ ^17^ ^]^ which highlighted the need to investigate whether immune cells mediate the OMD expression of hRIFs. In terms of apoptosis, Hamaya et al. reported the regulative role of OMD in the regulation of apoptosis and growth in osteoblast cells.^[^ ^50^ ^]^ Given the well‐established role of apoptosis in oxidative stress‐related renal tubular injury^[^ ^58^, ^59^ ^]^ and interstitial biomineralization,^[^ ^18^ ^]^ it is reasonable to further clarify the potential role of OMD‐mediated cell apoptosis in RP formation.

Based on previous studies,^[^ ^60^, ^61^ ^]^ we utilized a high hydroxyproline diet and subcutaneous injection of VD_2_ to establish mouse models with CaOx nephrocalcinosis. Consistent with prior findings,^[^ ^60^, ^61^ ^]^ CaOx crystal deposits predominantly appeared in renal tubules. The transcytosis of intratubular crystals from the apical to the basal aspect of the epithelium may contribute to RP formation.^[^ ^60^, ^62^ ^]^ Additionally, we observed upregulated ossification‐related proteins in mouse kidneys, which partially simulated osteogenic‐like microenvironment in RP tissues.^[^ ^9^, ^17^, ^25^ ^]^ We further generated mice with conditional *Omd* knockout in renal fibroblasts, and found that *Col1a2‐CreERT^tg/+^*; *Omd^flox/flox^* mice showed reduced CaOx nephrocalcinosis, particularly in intratubular crystal deposits. This could be partially explained by several factors. First, a recent study delicately demonstrated that macrophages made transepithelial migration into the tubular lumen to clear intratubular particles, maintaining unobstructed tubular systems.^[^ ^63^ ^]^ Another study showed that renal interstitial collagen was upregulated in rats with CaOx nephrocalcinosis.^[^ ^60^ ^]^ Omd might enhance renal interstitial collagen, which might serve as a barrier obstructing the transepithelial migration of macrophages and preventing the clearance of intratubular CaOx crystals. Second, the secreted Omd might mediate crosstalk between interstitial and tubular epithelial cells, promoting the adherence of CaOx crystals to the luminal surfaces. It will be of importance and interest to test the above hypothesis. On the other hand, considering that renal interstitial crystals were age or induction‐time dependent in mouse models,^[^ ^64^ ^]^ further studies are warranted to clarify whether interstitial crystal deposits decrease in *Col1a2‐CreERT^tg/+^*; *Omd^flox/flox^* mice with a prolonged time of induction. Nevertheless, there remains in lack of established animal models ideally simulating the pathophysiological process of RP formation.^[^ ^65^ ^]^ Ideal animal models coupled with the lineage tracing of fibroblasts are expected to help understand how phenotypic transitions of hRIFs contribute to RP formation.

Combined our findings in vitro and in vivo, we identified OMD as a novel marker for renal interstitial biomineralization within RP tissues, and highlighted that OMD promoted osteogenic‐like differentiation of hRIFs and thus induced osteogenic‐like microenvironment to participate in RP formation. Our study not only enriched the evidence of OMD involved in ectopic calcifications,^[^ ^45^, ^46^ ^]^ but also comprehensively revealed the mechanism of RP formation from the perspective of the osteogenic‐like microenvironment. Nevertheless, we have to acknowledge the main limitations. First, utilizing the content of Ca and P elements, we calculated a Ca/P ratio of ≈10:2.6 in the core region of CaOx. This ratio exceeds the typical 10:6 ratio of hydroxyapatite, which is reported as the main composition of RPs,^[^ ^66^ ^]^ likely due to a mixture of hydroxyapatite with CaOx or CaCO_3_ in the core region of CaOx stones. This study could have accurately analyzed the composition using X‐ray diffraction or Fourier transform infrared spectroscopy. Second, given that OMD is a secreted ECM protein,^[^ ^41^ ^]^ our study did not investigate its role in the interaction or crosstalk between hRIFs and other renal cells. Third, we focused on the underlying mechanism of OMD in RP formation, and it is also important to clarify the etiology driving the disruption of OMD. Fourth, our results of animal models should be interpreted with caution. Given *Col1a2*‐*CreERT^tg/+^*; *Omd* ^f^ *^lox/flox^* mice might generate the deletion of *Omd* in total fibroblasts, we cannot rule out the effect of *Omd* deletion in fibroblasts of other organs on CaOx nephrocalcinosis. Organ and cell type‐dual specific targeting can be achieved by utilizing dual recombination systems based on Dre‐rox and Cre‐loxP,^[^ ^67^, ^68^ ^]^ and further investigations utilizing an animal model with dual recombination systems are required to verify our findings. Additionally, mice possess a unipapillary system,^[^ ^65^ ^]^ unlike the multipapillary system found in humans, and the etiology of RP plaques is diverse, including but not limited to abnormal urinary constituents.^[^ ^53^ ^]^ The advancement of experimental research on RP formation has faced significant hindrances primarily attributed to the absence of ideal established animal models. Further investigations utilizing an ideal animal model are required to comprehensively elucidate the role of OMD in RP formation.

## 4 Conclusions

Our study for the first time identified OMD as a potential marker for renal interstitial biomineralization in RP tissues, and revealed a novel feedback loop of OMD/BMP2/BMPR1A/RUNX2/OMD driving hRIFs to adopt osteogenic‐like fates, by which OMD induced osteogenic‐like microenvironment to participate in RP formation. Our findings provided new insights into clarifying RP formation from the perspective that osteogenic‐like microenvironment played a vital role in CaP deposits within renal interstitium. Nevertheless, further investigations utilizing an ideal animal model are required to comprehensively elucidate the role of OMD in RP formation, which is promising to offer a theoretical basis for the development of preventive drugs for CaOx stones.

## 5 Experimental Section

### Clinical Samples

After obtaining approval (Proof Number: 202103089) from Xiangya Hospital Ethics Committee and written informed consent from patients before surgery, renal papillae, and renal medulla tissues were collected from patients undergoing nephrectomy due to renal cancers, as performed in the previous study.^[^ ^15^ ^]^ The inclusion criteria for patients were as follows: 1) absence of renal dysfunction; 2) absence of hydronephrosis or mild hydronephrosis; 3) absence of renal atrophy determined by computed tomography (CT), and 4) the dissected renal papilla was more than 3 cm away from the edge of the tumor. Normal renal papillae were defined as the patient had no history of urolithiasis and Von‐Kossa staining showed no calcium depositions in renal interstitium within renal papillae. Renal papillae with RP were defined as that the patient had CaOx kidney stones or had a history of CaOx stones, and Von‐Kossa staining showed calcium deposits in renal interstitium. In this study, 28 NRP tissues from 22 patients and 31 RP tissues from 24 patients were obtained in the institution between April 2021 and March 2024. The clinical characteristics of the included patients were summarized in Table S1 of the Supporting Information.

### Cell Transfection

As performed in the previous study,^[^ ^19^ ^]^ reconstituted lentivirus (GenePharma, China) was transfected to overexpress *OMD* (Len‐*OMD*) or BMP2 (Len‐*BMP2*), and silence OMD (Len‐sh‐*OMD*), BMP2 (Len‐sh‐*BMP2*) or RUNX2 (Len‐sh‐*RUNX2*) in hRIFs. Lentivirus carrying a scrambled sequence was used as the negative control (Len‐ctrl; Len‐sh‐ctrl). siRNA (RiboBio, China) was used to silence NEDD4 with Lipo2000 (Invitrogen, USA). The targeted sequences designed for sh‐RNA or si‐RNA were listed in Table S2 of the Supporting Information. Additionally, reconstituted plasmids (GenePharma, China) were transfected in hRIFs to express Flag‐*OMD* or HA‐*BMP2* with Lipo2000.

### RNA‐Seq

hRIFs were cultured either in normal medium or in osteogenic medium for one week. Total RNA was then extracted to create cDNA libraries, which were subsequently subjected to RNA sequencing using the Illumina platform (HiSeq X Ten, RiboBio Co., Ltd., China). The datasets of RNA‐seq are available in the GEO DataSets (GSE203110).

### Reverse Transcription and qRT‐PCR

RNA was extracted from hRIFs or tissues using the SteadyPure Universal RNA Extraction Kit (AG, China), followed by cDNA synthesis using the PrimeScript RT Reagent Kit (Takara, Japan). Subsequently, qRT‐PCR (QuantStudio5/7, USA) was performed using the SYBR Green PCR Kit (Takara, Japan). Relative gene expressions, normalized to *GAPDH*, were analyzed using the 2^−ΔΔCt^ method, with primer sequences provided in Table S3 of the Supporting Information.

### Immunoblotting (IB)

IB was performed as in the previous study.^[^ ^19^ ^]^ In brief, protein blots were visualized using chemiluminescence in a ChemiDoc XRS system (Bio‐Rad, USA), with details of primary and secondary antibodies provided in Table S4 of the Supporting Information. The intensity of protein blots was quantified using ImageJ software and normalized to that of GAPDH, expressed as the ratio relative to the control group.

### Co‐IP

The antibody for a targeted protein was conjugated to magnetic protein A/G beads (MCE, USA) at room temperature for 1 h, and the cell lysate supernatant was incubated with the beads at 4 °C overnight. Following washes with lysis buffer, the precipitated complexes were subjected to proteomics by mass spectrometry or IB. Proteomics by mass spectrometry was performed on an MALDI‐TOF instrument (Bruker Daltonics, USA) by OE Biotech Co., Ltd. (Shanghai, China) as described.^[^ ^69^ ^]^

### Immunofluorescence

ECM of hRIFs was prepared by repeated freeze‐thaw cycles. Immunofluorescence of ECM, hRIFs, and renal tissues was performed as reported in the previous study.^[^ ^14^ ^]^ Multiplex immunofluorescence tyramide signal amplification assays^[^ ^70^ ^]^ were performed to costain Col1a2, Cre, Omd, and Bmp2 in mouse kidneys. Initially, antigen retrieval was performed with Antigen Unmasking Solution (Vector Laboratories, USA), followed by the suppression of endogenous peroxidase activity by 5% hydrogen peroxide. After blocking with 10% goat serum (Solarbio, China), the slide was incubated with primary antibodies against Col1a2, followed by the incubation with HRP‐conjugated immunoglobulin G and treatment with iF488‐Tyramide. After removing antibodies with Antigen Unmasking Solution, the remaining proteins were sequentially stained with iF594‐Tyramide and iF647‐Tyramide, following the same procedure of antibody removal and incubation cycles. Finally, DAPI (Servicebio, China) was added, and the panoramic image was obtained with a slide scanner (3DHISTECH, Hungary). The colocalization ratio of objects in dual‐color confocal images was measured by the confocal microscope system (Leica TCS SP8 X, Germany) or by ImageJ software in accordance with a practical guide reported by Dunn et al.^[^ ^71^ ^]^ Additionally, the details of antibodies were listed in Table S4 of the Supporting Information.

### ChIP

ChIP was performed using EpiQuik ChIP Kit (Epigentek, USA) in accordance with the previous study.^[^ ^14^ ^]^ The immunoprecipitated products underwent q‐PCR analysis and subsequent verification via 1% agarose gel electrophoresis. Positive controls included anti‐RNA polymerase II (RPII) and primers targeting the *GAPDH* promoter regions, while normal mouse IgG served as the negative control. Table S5 of the Supporting Information provided the primer details for amplifying *OMD* promoter regions with predicted RUNX2 or SMAD3 binding sites (Table S6, Supporting Information).

### SEM and TEM

SEM and TEM were performed on Quanta‐200 (FEI, USA) and Tecnai G2 F20 (FEI, USA), respectively, as described in the previous study.^[^ ^72^ ^]^ For immunoelectron microscopic analysis, samples were prepared in accordance with a similar study reported by Evan et al.^[^ ^11^ ^]^ Briefly, nickel grids carrying ultrathin sections were blocked with 1% bovine serum albumin, followed by incubation with primary antibodies with IgG as the negative control. Subsequently, 10 nm colloidal gold‐conjugated secondary antibodies (Servicebio, China) were added. Uranium counterstaining was performed by immersing the nickel grids in a solution of 2% uranyl acetate saturated in ethanol. After washing with 70% ethanol and then ultrapure water, samples were dried for TEM analysis. The 10 nm sized black gold particles represent the positive expression of targeted antigens.

### Subcutaneous Ectopic Implantation of Osteogenic‐Induced hRIFs In Vivo

After obtaining approval from the Institutional Experimental Animal Committee of Central South University (Proof Number: CSU‐2022‐0022), 21 eight‐week‐old female nude mice, sourced from the Animal Experimental Center of Central South University, were utilized in this study. Diligent efforts were taken to minimize suffering and reduce the number of mice used. hRIFs were induced with osteogenic medium for 14 days before implantation. Subsequently, 1 × 10^7^ cells were seeded onto 8.5 mm × 5 mm × 1.5 mm hydroxyapatite (HA) scaffolds (Hengtian Biotechnology, China). Grafts were implanted subcutaneously on the dorsal surface of mice, with one graft placed on each side, under general anesthesia. To evaluate the survival of hRIFs, HA scaffolds carrying hRIFs transfected with lentivirus encoding luciferase were implanted. After eight weeks, fluorescence was assessed using animal fluorescence imaging (IVIS Spectrum, PerkinElmer, USA) 15 min postinjection of d‐Luciferin (YEASEN, China). To evaluate the role of OMD in the osteogenic‐like phenotype of hRIFs, six groups of grafts were implanted subcutaneously following the same procedure, including grafts without cells; grafts with hRIFs transfected with nothing, Len‐ctrl, Len‐*OMD*, Len‐sh‐ctrl or Len‐sh‐*OMD*. Eight weeks after transplantation, grafts were collected and fixed with 4% paraformaldehyde. Slides of grafts were prepared as described by the previous study,^[^ ^19^ ^]^ and the staining of HE, Masson's trichrome as well as IHC was performed.

### Animal Models with CaOx Nephrocalcinosis

After obtaining approval from the Institutional Experimental Animal Committee of Central South University (Proof Number: CSU‐2022‐0454). *Omd^flox/flox^* mice *and Col1a2‐CreERT^tg/+^* mice were purchased from Cyagen Biosciences Co., Ltd. (China). The design of *Omd^flox/flox^* was described in Methods section of the Supporting Information, and genotypes were determined by PCR using primers listed in Table S7 of the Supporting Information. *Col1a2‐CreERT^tg/+^* mice contain tamoxifen‐inducible Cre under the control of the proα2(I) collagen promoter (*Col1a2*). *Omd^flox/flox^* female mice and *Col1a2‐CreERT^tg/+^* male mice were crossed to obtain *Col1a2‐CreERT^tg/+^*; *Omd^+/flox^* male mice, followed by crossing with *Omd^flox/flox^* female mice to generate *Col1a2‐CreERT^tg/+^*; *Omd^flox/flox^* mice. *Col1a2‐CreERT^tg/+^*; *Omd^flox/flox^* mice, along with their littermate controls (*Omd^flox/flox^*) were injected with 4‐OH tamoxifen (16 mg/100 g mouse) intraperitoneally from 5 to 11 weeks of age, every other day. From 7 to 11 weeks of age, mice received a 5% hydroxyproline diet (18% protein; 1% Ca; 0.7% Pi) and subcutaneous injection of VD_2_ (150IU/100 g) to induce CaOx nephrocalcinosis,^[^ ^60^, ^61^, ^73^ ^]^ and the control group received a normal diet (18% protein; 1% Ca; 0.7% Pi) and subcutaneous injection of saline. Four weeks after treatment, mice were euthanized and then kidneys were collected.

### Statistical Analysis

Each experiment was independently replicated at least three times. Categorical variables were analyzed using the Chi‐squared test or Fisher's exact test; quantitative data, presented as mean ± SD, were analyzed using one‐way ANOVA or unpaired Student's *t*‐test, as appropriate. Spearman's rank correlation was employed to assess associations between two parameters. A two‐tailed *P*‐value ≤ 0.05 was considered statistically significant. Statistical analyses were performed using GraphPad Prism 8 software (GraphPad Software, USA) and R software (version 4.0.2). In all analyses, *, **, and *** indicated *P*‐values of < 0.05, < 0.01, and < 0.001, respectively.

### Ethical Statement

Ethics approval was granted by the Xiangya Hospital Ethics Committee (Approval Number: 202103089) and the Institutional Experimental Animal Committee of Central South University (Approval Number: CSU‐2022‐0022; CSU‐2022‐0454). Written informed consent for surgical procedures, sample collection, and the anonymous publication of clinical characteristics was obtained from all patients before surgery.

## Conflict of Interest

The authors declare no conflict of interest.

## Author Contributions

Z.Z., H.C., T.L., F.Z., and Y.C. designed the experiments. Z.Z., F.H., M.G., M.L., Y.Z. L.T., J.W., and H.Y. performed the biochemical experiments. Z.Z., F.H., M.G., M.L., J.W., H.Y., C.H., and J.C. performed the animal experiments. Z.Z., C.H., J.C., Z.Y., Z.C., and Y.L. performed the pathological experiments. Z.Z., F.Z., Y.C., and T.L. analyzed the data. Z.Z. and T.L. wrote the paper. H.C., T.L., F.Z., and Y.C. revised the manuscript and supervised the project. The manuscript has been read and approved by all authors.

## Supporting information

Supporting Information

Supporting Table

## Data Availability

The RNA‐seq datasets generated during this study are available in the GEO DataSets (GSE203110). Proteomics data obtained by mass spectrometry are provided in the Supporting Information. Other datasets used and/or analyzed during this study are available from the corresponding author upon reasonable request.

## References

[1] K. Stamatelou , D. S. Goldfarb , Healthcare 2023, 11, 424. 36766999 10.3390/healthcare11030424 PMC9914194

[2] C. Fisang , R. Anding , S. C. Müller , S. Latz , N. Laube , Dtsch. Arztebl. Int. 2015, 112, 83. 25721435 10.3238/arztebl.2015.0083 PMC4349965

[3] S. Tan , D. Yuan , H. Su , W. Chen , S. Zhu , B. Yan , F. Sun , K. Jiang , J. Zhu , BJU Int. 2024, 133, 34. 37696625 10.1111/bju.16179

[4] B. H. Eisner , D. S. Goldfarb , J. Am. Soc. Nephrol. 2014, 25, 2685. 25104802 10.1681/ASN.2014060631 PMC4243365

[5] S. R. Khan , M. S. Pearle , W. G. Robertson , G. Gambaro , B. K. Canales , S. Doizi , O. Traxer , H. G. Tiselius , Nat. Rev. Dis. Primers 2016, 2, 16008. 27188687 10.1038/nrdp.2016.8 PMC5685519

[6] A. Randall , Ann. Surg. 1937, 105, 1009. 17856988 10.1097/00000658-193706000-00014 PMC1390483

[7] M. Daudon , D. Bazin , E. Letavernier , Urolithiasis 2015, 43, 5. 10.1007/s00240-014-0703-y 25098906

[8] H. J. Chung , Transl. Androl. Urol. 2014, 3, 251. 26816774 10.3978/j.issn.2223-4683.2014.07.03 PMC4708577

[9] C. Gay , E. Letavernier , M. C. Verpont , M. Walls , D. Bazin , M. Daudon , N. Nassif , O. Stephan , M. de Frutos , ACS Nano 2020, 14, 1823. 31909991 10.1021/acsnano.9b07664

[10] S. R. Khan , D. E. Rodriguez , L. B. Gower , M. Monga , J. Urol. 2012, 187, 1094. 22266007 10.1016/j.juro.2011.10.125 PMC3625933

[11] A. P. Evan , F. L. Coe , S. R. Rittling , S. M. Bledsoe , Y. Shao , J. E. Lingeman , E. M. Worcester , Kidney Int. 2005, 68, 145. 15954903 10.1111/j.1523-1755.2005.00388.x

[12] A. P. Evan , F. L. Coe , J. E. Lingeman , E. Worcester , Urol. Res. 2005, 33, 383. 16078085 10.1007/s00240-005-0488-0

[13] A. P. Evan , F. L. Coe , J. E. Lingeman , Y. Shao , A. J. Sommer , S. B. Bledsoe , J. C. Anderson , E. M. Worcester , Anat. Rec. (Hoboken) Evol. Biol. 2007, 290, 1315. 10.1002/ar.20580 17724713

[14] Z. Zhu , F. Huang , Y. Jiang , S. Ruan , M. Liu , Y. Zhang , Y. Li , J. Chen , Y. Cui , Z. Chen , H. Chen , F. Zeng , Mol. Med. 2022, 28, 162. 36581839 10.1186/s10020-022-00576-4 PMC9798568

[15] Z. Zhu , X. Zhang , Y. Jiang , S. Ruan , F. Huang , H. Zeng , M. Liu , W. Xia , F. Zeng , J. Chen , Y. Cui , H. Chen , Epigenomics 2021, 13, 1171. 34325517 10.2217/epi-2021-0212

[16] A. Evan , J. Lingeman , F. L. Coe , E. Worcester , Kidney Int. 2006, 69, 1313. 16614720 10.1038/sj.ki.5000238

[17] V. H. Canela , W. S. Bowen , R. M. Ferreira , F. Syed , J. E. Lingeman , A. R. Sabo , D. Barwinska , S. Winfree , B. B. Lake , Y.‐H. Cheng , J. P. Gaut , M. Ferkowicz , K. A. LaFavers , K. Zhang , F. L. Coe , E. Worcester , S. Jain , M. T. Eadon , J. C. Williams , T. M. El‐Achkar , Nat. Commun. 2023, 14, 4140. 37468493 10.1038/s41467-023-38975-8 PMC10356953

[18] G. Priante , M. Ceol , L. Gianesello , C. Furlan , D. Del Prete , F. Anglani , Cell Death Discovery 2019, 5, 57. 30701089 10.1038/s41420-019-0138-x PMC6349935

[19] Z. Zhu , S. Ruan , Y. Jiang , F. Huang , W. Xia , J. Chen , Y. Cui , C. He , F. Zeng , Y. Li , Z. Chen , H. Chen , Cell. Mol. Life Sci. 2021, 78, 7831. 34724098 10.1007/s00018-021-03972-x PMC11071709

[20] Z. Wang , Y. Zhang , J. Zhang , Q. Deng , H. Liang , Int. J. Mol. Med. 2021, 48, 149. 34132361 10.3892/ijmm.2021.4982 PMC8208620

[21] A. P. Evan , F. L. Coe , J. Lingeman , S. Bledsoe , E. M. Worcester , Am. J. Physiol.: Renal. Physiol. 2018, 315, F1236. 30066583 10.1152/ajprenal.00035.2018 PMC6293286

[22] C. Verrier , D. Bazin , L. Huguet , O. Stephan , A. Gloter , M. C. Verpont , V. Frochot , J. P. Haymann , I. Brocheriou , O. Traxer , M. Daudon , E. Letavernier , J. Urol. 2016, 196, 1566. 27157373 10.1016/j.juro.2016.04.086

[23] E. R. Taylor , M. L. Stoller , Urolithiasis 2015, 43, 41. 10.1007/s00240-014-0718-4 25475492

[24] R. C. Haggitt , J. A. Pitcock , J. Urol. 1971, 106, 342. 4106437 10.1016/s0022-5347(17)61284-9

[25] S. R. Khan , G. Gambaro , Anat. Rec. 2016, 299, 5. 10.1002/ar.23275 26414710

[26] J. Myung , K. B. Kim , C. M. Crews , Med. Res. Rev. 2001, 21, 245. 11410931 10.1002/med.1009 PMC2556558

[27] N. Zheng , N. Shabek , Annu. Rev. Biochem. 2017, 86, 129. 28375744 10.1146/annurev-biochem-060815-014922

[28] J. Chen , Y. M. Dang , M. C. Liu , L. Gao , T. Guan , A. Hu , L. Xiong , H. Lin , Biochim. Biophys. Acta, Mol. Cell. Res. 2024, 1871, 119771. 38844181 10.1016/j.bbamcr.2024.119771

[29] J. Kushioka , T. Kaito , R. Okada , H. Ishiguro , Z. Bal , J. Kodama , R. Chijimatsu , M. Pye , M. Narimatsu , J. L. Wrana , Y. Inoue , H. Ninomiya , S. Yamamoto , T. Saitou , H. Yoshikawa , T. Imamura , Bone Res. 2020, 8, 41. 33298874 10.1038/s41413-020-00115-z PMC7680794

[30] B. G. Kim , J. H. Lee , J. Yasuda , H. M. Ryoo , J. Y. Cho , J. Bone Miner. Res. 2011, 26, 1411. 21308777 10.1002/jbmr.348

[31] J. Bonor , E. L. Adams , B. Bragdon , O. Moseychuk , K. J. Czymmek , A. Nohe , J. Cell. Physiol. 2012, 227, 2880. 21938723 10.1002/jcp.23032 PMC3310286

[32] T. L. Bailey , M. Boden , F. A. Buske , M. Frith , C. E. Grant , L. Clementi , J. Ren , W. W. Li , W. S. Noble , Nucleic Acids Res. 2009, 37, W202. 19458158 10.1093/nar/gkp335 PMC2703892

[33] O. Fornes , J. A. Castro‐Mondragon , A. Khan , R. van der Lee , X. Zhang , P. A. Richmond , B. P. Modi , S. Correard , M. Gheorghe , D. Baranašić , W. Santana‐Garcia , G. Tan , J. Chèneby , B. Ballester , F. Parcy , A. Sandelin , B. Lenhard , W. W. Wasserman , A. Mathelier , Nucleic Acids Res. 2020, 48, D87. 31701148 10.1093/nar/gkz1001 PMC7145627

[34] I. V. Kulakovskiy , I. E. Vorontsov , I. S. Yevshin , R. N. Sharipov , A. D. Fedorova , E. I. Rumynskiy , Y. A. Medvedeva , A. Magana‐Mora , V. B. Bajic , D. A. Papatsenko , F. A. Kolpakov , V. J. Makeev , Nucleic Acids Res. 2018, 46, D252. 29140464 10.1093/nar/gkx1106 PMC5753240

[35] C. E. Grant , T. L. Bailey , W. S. Noble , Bioinformatics 2011, 27, 1017. 21330290 10.1093/bioinformatics/btr064 PMC3065696

[36] L. Zhang , G. Jiao , S. Ren , X. Zhang , C. Li , W. Wu , H. Wang , H. Liu , H. Zhou , Y. Chen , Stem Cell Res. Ther. 2020, 11, 38. 31992369 10.1186/s13287-020-1562-9 PMC6986095

[37] X. Luo , H. M. Chang , Y. Yi , P. C. K. Leung , Y. Sun , FASEB J. 2021, 35, 21845. 10.1096/fj.202100670RR 34369625

[38] M. L. Zou , Z. H. Chen , Y. Y. Teng , S. Y. Liu , Y. Jia , K. W. Zhang , Z. L. Sun , J. J. Wu , Z. D. Yuan , Y. Feng , X. Li , R. S. Xu , F. L. Yuan , Front. Mol. Biosci. 2021, 8, 593310. 34026818 10.3389/fmolb.2021.593310 PMC8131681

[39] N. L. Miller , J. C. Williams, Jr. , A. P. Evan , S. B. Bledsoe , F. L. Coe , E. M. Worcester , L. C. Munch , S. E. Handa , J. E. Lingeman , BJU Int. 2010, 105, 242. 19549258 10.1111/j.1464-410X.2009.08637.x PMC2807918

[40] B. R. Matlaga , J. C. Williams , S. C. Kim , R. L. Kuo , A. P. Evan , S. B. Bledsoe , F. L. Coe , E. M. Worcester , L. C. Munch , J. E. Lingeman , J. Urol. 2006, 175, 1720. 16600740 10.1016/S0022-5347(05)01017-7

[41] M. Wendel , Y. Sommarin , D. Heinegård , J. Cell Biol. 1998, 141, 839. 9566981 10.1083/jcb.141.3.839 PMC2132750

[42] F. Zhu , M. S. Friedman , W. Luo , P. Woolf , K. D. Hankenson , J. Cell. Physiol. 2012, 227, 2677. 21898406 10.1002/jcp.23010 PMC3241898

[43] W. Lin , L. Gao , W. Jiang , C. Niu , K. Yuan , X. Hu , R. Ma , Z. Huang , BMC Oral Health 2019, 19, 22. 30670012 10.1186/s12903-018-0680-6 PMC6341608

[44] W. Lin , X. Zhu , L. Gao , M. Mao , D. Gao , Z. Huang , Cell Death Dis. 2021, 12, 147. 33542209 10.1038/s41419-021-03404-5 PMC7862363

[45] N. T. Skenteris , T. Seime , A. Witasp , E. Karlöf , G. B. Wasilewski , M. A. Heuschkel , A. M. G. Jaminon , L. Oduor , R. Dzhanaev , M. Kronqvist , M. Lengquist , F. Peeters , M. Söderberg , R. Hultgren , J. Roy , L. Maegdefessel , H. Arnardottir , E. Bengtsson , I. Goncalves , T. Quertermous , C. Goettsch , P. Stenvinkel , L. J. Schurgers , L. Matic , Clin. Transl. Med. 2022, 12, 682. 10.1002/ctm2.682 PMC8858609 35184400

[46] I. Gonçalves , L. Oduor , F. Matthes , N. Rakem , J. Meryn , N. T. Skenteris , A. Aspberg , M. Orho‐Melander , J. Nilsson , L. Matic , A. Edsfeldt , J. Sun , E. Bengtsson , Stroke 2022, 53, 79. 35135320 10.1161/STROKEAHA.121.037223

[47] V. Rosen , Cytokine Growth Factor Rev. 2009, 20, 475. 19892583 10.1016/j.cytogfr.2009.10.018

[48] M. Lucchini , M. L. Couble , A. Romeas , M. J. Staquet , F. Bleicher , H. Magloire , J. C. Farges , J. Dent. Res. 2004, 83, 552. 15218045 10.1177/154405910408300708

[49] F. Posa , E. H. Baha‐Schwab , Q. Wei , A. Di Benedetto , S. Neubauer , F. Reichart , H. Kessler , J. P. Spatz , C. Albiges‐Rizo , G. Mori , E. A. Cavalcanti‐Adam , Biomaterials 2021, 267, 120484. 33142116 10.1016/j.biomaterials.2020.120484

[50] E. Hamaya , T. Fujisawa , M. Tamura , Int. J. Mol. Med. 2019, 44, 2336. 31638177 10.3892/ijmm.2019.4376

[51] M. L. Stoller , M. V. Meng , H. M. Abrahams , J. P. Kane , J. Urol. 2004, 171, 1920. 15076312 10.1097/01.ju.0000120291.90839.49

[52] R. O. Weller , B. Nester , S. A. Cooke , J. Pathol. 1972, 107, 211. 5084933 10.1002/path.1711070308

[53] S. R. Khan , B. K. Canales , P. R. Dominguez‐Gutierrez , Nat. Rev. Nephrol. 2021, 17, 417. 33514941 10.1038/s41581-020-00392-1

[54] A. Chidambaram , D. Rodriguez , S. Khan , L. Gower , Urolithiasis 2015, 43, 77. 10.1007/s00240-014-0704-x PMC4285617 25119505

[55] T. Tashima , S. Nagatoishi , J. M. M. Caaveiro , M. Nakakido , H. Sagara , O. Kusano‐Arai , H. Iwanari , H. Mimuro , T. Hamakubo , S. I. Ohnuma , K. Tsumoto , Commun. Biol. 2018, 1, 33. 30271919 10.1038/s42003-018-0038-2 PMC6123635

[56] K. Taguchi , S. Hamamoto , A. Okada , R. Unno , H. Kamisawa , T. Naiki , R. Ando , K. Mizuno , N. Kawai , K. Tozawa , K. Kohri , T. Yasui , J. Am. Soc. Nephrol. 2017, 28, 333. 27297950 10.1681/ASN.2015111271 PMC5198277

[57] K. Ninomiya , T. Miyamoto , J. Imai , N. Fujita , T. Suzuki , R. Iwasaki , M. Yagi , S. Watanabe , Y. Toyama , T. Suda , Biochem. Biophys. Res. Commun. 2007, 362, 460. 17714690 10.1016/j.bbrc.2007.07.193

[58] Q. L. Ye , D. M. Wang , X. Wang , Z. Q. Zhang , Q. X. Tian , S. Y. Feng , Z. H. Zhang , D. X. Yu , D. M. Ding , D. D. Xie , Chem. Biol. Interact. 2021, 347, 109605. 34333021 10.1016/j.cbi.2021.109605

[59] Q. L. Peng , C. Y. Li , Y. W. Zhao , X. Y. Sun , H. Liu , J. M. Ouyang , Oxid. Med. Cell. Longevity 2021, 2021, 6463281. 10.1155/2021/6463281 PMC7946465 33763169

[60] S. Joshi , W. L. Clapp , W. Wang , S. R. Khan , Biochim. Biophys. Acta 2015, 1852, 2000. 26122267 10.1016/j.bbadis.2015.06.020 PMC4523408

[61] Z. Jia , S. Wang , J. Tang , D. He , L. Cui , Z. Liu , B. Guo , L. Huang , Y. Lu , H. Hu , Urology 2014, 83, 509.e7. 10.1016/j.urology.2013.11.004 24468523

[62] V. Kumar , G. Farell , S. Yu , S. Harrington , L. Fitzpatrick , E. Rzewuska , V. M. Miller , J. C. Lieske , J. Invest. Med. 2006, 54, 412. 10.2310/6650.2006.06021 17169263

[63] J. He , Y. Cao , Q. Zhu , X. Wang , G. Cheng , Q. Wang , R. He , H. Lu , Y. Weng , G. Mao , Y. Bao , J. Wang , X. Liu , F. Han , P. Shi , X. Z. Shen , Immunity 2024, 57, 106. 38159573 10.1016/j.immuni.2023.12.003

[64] Y. Liu , L. Mo , D. S. Goldfarb , A. P. Evan , F. Liang , S. R. Khan , J. C. Lieske , X. R. Wu , Am. J. Physiol.: Renal. Physiol. 2010, 299, F469. 20591941 10.1152/ajprenal.00243.2010 PMC2944300

[65] D. T. Tzou , K. Taguchi , T. Chi , M. L. Stoller , Int. J. Surg. 2016, 36, 596. 27840313 10.1016/j.ijsu.2016.11.018

[66] A. P. Evan , J. E. Lingeman , F. L. Coe , J. H. Parks , S. B. Bledsoe , Y. Z. Shao , A. J. Sommer , R. F. Paterson , R. L. Kuo , M. Grynpas , J. Clin. Invest. 2003, 111, 607. 12618515 10.1172/JCI17038 PMC151900

[67] Y. Li , Z. Liu , X. Han , F. Liang , Q. Zhang , X. Huang , X. Shi , H. Huo , M. Han , X. Liu , H. Zhu , L. He , L. Shen , X. Hu , J. Wang , Q. D. Wang , N. Smart , B. Zhou , B. He , Circulation 2024, 149, 135. 38084582 10.1161/CIRCULATIONAHA.123.064301

[68] W. Pu , L. He , X. Han , X. Tian , Y. Li , H. Zhang , Q. Liu , X. Huang , L. Zhang , Q. D. Wang , Z. Yu , X. Yang , N. Smart , B. Zhou , Circ. Res. 2018, 123, 86. 29764841 10.1161/CIRCRESAHA.118.312981 PMC6015762

[69] J. Zheng , X. Huang , W. Tan , D. Yu , Z. Du , J. Chang , L. Wei , Y. Han , C. Wang , X. Che , Y. Zhou , X. Miao , G. Jiang , X. Yu , X. Yang , G. Cao , C. Zuo , Z. Li , C. Wang , S. T. Cheung , Y. Jia , X. Zheng , H. Shen , C. Wu , D. Lin , Nat. Genet. 2016, 48, 747. 27213290 10.1038/ng.3568

[70] E. R. Parra , M. Jiang , L. Solis , B. Mino , C. Laberiano , S. Hernandez , S. Gite , A. Verma , M. Tetzlaff , C. Haymaker , A. Tamegnon , J. Rodriguez‐Canales , C. Hoyd , C. Bernachez , I. Wistuba , Cancers 2020, 12, 255. 31972974 10.3390/cancers12020255 PMC7072187

[71] K. W. Dunn , M. M. Kamocka , J. H. McDonald , Am. J. Physiol.: Cell. Physiol. 2011, 300, C723. 21209361 10.1152/ajpcell.00462.2010 PMC3074624

[72] Z. Zhu , F. Huang , W. Xia , H. Zeng , M. Gao , Y. Li , F. Zeng , C. He , J. Chen , Z. Chen , Y. Li , Y. Cui , H. Chen , Front. Cell. Dev. Biol. 2020, 8, 596363. 33505960 10.3389/fcell.2020.596363 PMC7829506

[73] E. Bouderlique , E. Tang , J. Perez , A. Coudert , D. Bazin , M. C. Verpont , C. Duranton , I. Rubera , J. P. Haymann , G. Leftheriotis , L. Martin , M. Daudon , E. Letavernier , Am. J. Pathol. 2019, 189, 2171. 31449775 10.1016/j.ajpath.2019.07.013

