# Supplementary material for: Osteogenic‐Like Microenvironment of Renal Interstitium Induced by Osteomodulin Contributes to Randall's Plaque Formation
Source: Adv Sci (Weinh). 2024 Sep 3;11(40):2405875. doi: 10.1002/advs.202405875 (PMC11516157; doi:10.1002/advs.202405875)
Supplement: Supplementary file 1 — Supporting Information [file ADVS-11-2405875-s001.docx]

**Supplementary materials for “Osteogenic-like micro-environment of renal interstitium induced by Osteomodulin contributes to Randall’s plaque formation”**

**Supplementary methods**

**Cell isolation and culture**

human renal interstitial fibroblasts (hRIFs) were isolated from renal medulla and papillae as described by our previous study^[1]^. As performed by Beijnum et. al^[2]^, human renal peritubular capillary endothelial cells (hRPCECs) were isolated from renal papillae using MicroBeads (Miltenyi Biotechnology, Germany) coated with anti-CD34 antibody. Similarly, human CLCNKA positive tubular epithelial cells (CLCNKA+ hTECs) and human AQP2 positive collecting duct cells (AQP2+ hCDCs) were isolated from renal papillae using MicroBeads coated with anti-CLCNKA antibody and anti-AQP2 antibody^[3]^, respectively. Primary cells between passages three and six were used for further experiments. Cells were cultured in DMEM (BI, Israel) supplemented with 10% fetal bovine serum (BI, Israel), 100 U/mL penicillin, and 100 μg/mL streptomycin (BI, Israel) at 37 °C in the presence of 5% CO_2_. Osteogenic differentiation was induced with DMEM medium containing dexamethasone (100 nM; Sigma, USA), ascorbic acid (200 μM; Sigma, USA), and β-glycerophosphate (10 mM; Sigma, USA), which is widely used as osteogenic medium in previous studies^[1, 4]^.

**Alizarin Red Staining (ARS) and** **alkaline phosphatase (ALP) activity assay**

As described^[5]^, calcium deposits in cell layers were stained with Alizarin Red (PH=4.0, Solarbio, China), and ARS in a stained monolayer was extracted with acetic acid and then neutralized with ammonium hydroxide, followed by semi-quantification with colorimetric detection at 405 nm. The ALP activity of cell lysates was assessed using an ALP colorimetric assay kit (Beyotime, China) according to the manufacturer’s instructions. Considering the differences in cell density among groups, ARS and ALP activity were normalized to the total protein of cell lysate determined by a BCA Protein Assay Kit (Beyotime, China).

**Immunohistochemistry (IHC)**, **hematoxylin and eosin (HE), Von Kossa and Masson's trichrome staining**

The preparation of slides and IHC staining were performed as in our previous study^[6]^.

The Von-Kossa Kit (Solaribo, China) was employed to stain calcium deposits in renal papillae according to the manufacturer’s guidelines, followed by counterstaining with HE). HE staining and Masson's trichrome staining were carried out using ready-to-use kits (Solaribo, China) according to the manufacturer’s instructions. As described by our and other studies^[6-8]^, semi-quantitative analysis of IHC staining density was carried out with the “IHC Toolbox” plugin in Image J^[9]^, and semi-quantitative analysis of Masson's trichrome staining was carried out with “color deconvolution” plugin in Image J^[10]^. All semi-quantitative analyses were performed with the blind.

**Generation of C57BL/6J *Omd^flox/flox^* mice**

*Omd^flox/flox^* mice were created by CRISPR/Cas-mediated genome engineering. The *Omd* gene (NCBI Reference Sequence: NM_012050; Ensembl: ENSMUSG00000048368) is located on mouse chromosome 13. Four exons are identified, with the ATG start codon in exon 3 and the TAG stop codon in exon 4 (Transcript: ENSMUST00000221170). Exon 3 was selected as the conditional knockout region (cKO region). Deletion of this region resulted in the loss of function of the mouse Omd gene. To engineer the targeting vector, homologous arms, and cKO region were generated by PCR using BAC clone RP23-9H18 as template. Cas9, gRNA, and targeting vector were co-injected into fertilized eggs for cKO mouse production. The F0 pups were genotyped by PCR followed by sequencing analysis. For further experiments, genotypes were determined by PCR using primers listed in Table S7.

**Supplementary Tables**

**Table S1.** The characteristics of included 46 patients undergoing nephrectomy due to renal cancers.

| Characteristics | NRP group  (n=22) | RP group (n=24) | *P* value |
| --- | --- | --- | --- |
| Age (years), mean±SD (range) | 54.6±8.4  (39-69) | 52.5±8.3  (37-68) | 0.38 |
| Gender (M/F) | 13/9 | 13/11 | 0.78 |
| Hydronephrosis degree (none/mild)*, n, % | 17/5 | 16/8 | 0.52 |
| Comorbidities, n (%) |  |  |  |
| Hypertension | 5 (22.7%) | 9 (37.5%) | 0.35 |
| Diabetes mellitus | 6(27.27%) | 8(33.33%) | 0.75 |
| Tumor size (cm), mean±SD (range) | 4.5±0.7  (3.4-6.1) | 4.7±0.8  (3.3-6.5) | 0.66 |

*The Society of Fetal Urology grading system was used to determine the hydronephrosis degree, and those with severe hydronephrosis were routinely excluded in the current study. M, male; F, female; SD, standard deviation. NRP, normal renal papillae; RP, renal papillae with Randall’s plaques.

**Table S2.** Targeted sequences designed for silencing *OMD*, *BMP2*, *RUNX2 or NEDD4*.

| Targeted Gene | Reconstituted lentivirus or siRNA | Targeted sequences (5’-3’) |
| --- | --- | --- |
| *OMD* | Len-sh1-*OMD* | CCATCATCAATGTACTGTGAT |
|  | Len-sh2-*OMD* | GAGTCAAAGTACATTGCCAAT |
|  | Len-sh3-*OMD* | CAAGGCATACAGATTCACAAA |
| *BMP2* | Len-sh1-*BMP2* | TCCAAGAGACATGTTAGGATA |
|  | Len-sh2-*BMP2* | AAGGCCATTGCTAGTAACTTT |
|  | Len-sh3-*BMP2* | AAACGTCAAGCCAAACACAAA |
| *RUNX2* | Len-sh1-*RUNX2* | TCAGCAGTGGCCCAGTGGTAT |
|  | Len-sh2-*RUNX2* | GTTTACTATTTAAGATGTACT |
|  | Len-sh3-*RUNX2* | TAATGACATTTGGACCCTTGA |
| *NEDD4* | si-*NEDD4* | TCCAATGATCTAGGGCCTTTA |

**Table S3.** Primer sequences designed for qRT-PCR.

| Gene | Forward primer 5′–3′ | Reverse primer 5′–3′ |
| --- | --- | --- |
| *GAPDH* | AACGTGTCAGTGGTGGACCTG | AGTGGGTGTCGCTGTTGAAGT |
| *OMD* | AGGCTGTGTCAGTGAATGCTT | TGGGATAGTCTTGAGTTTGCGAT |
| *RUNX2* | TGGTTACTGTCATGGCGGGTA | TCTCAGATCGTTGAACCTTGCTA |
| *BMP2* | ACCCGCTGTCTTCTAGCGT | TTTCAGGCCGAACATGCTGAG |
| *OGN* | TCTACACTTCTCCTGTTACTGCT | GAGGTAATGGTGTTATTGCCTCA |
| *EMILIN3* | GCCTCCCGCTACAGTCTCTA | CCATCTACACTGCCGGTATTC |
| *NPNT* | TGGCAAACTGTCAGTATGGCT | CTTGCAGATGTAGCTCCCAAA |
| *ADAMDEC1* | AGGAACAAGACCCAGCTAACC | AAAGGCATTATCCAGCACCAA |
| *CILP* | GCCCTGGTGAGTGGACAAC | GTCAGTGGTCCGAGCCTCTA |
| *VWA2* | ATGGGTCTAACAGCGTCGG | AGGAGTGGAACTGAACTGGAA |
| *DPT* | TGGGTGAATTTGAACCGGCAA | CGTAGTTCCATTGTCTGTCAGAA |
| *GPC3* | CCTTTGAAATTGTTGTTCGCCA | CCTGGGTTCATTAGCTGGGTA |
| *SERPINA3* | CCTGAAGGCCCCTGATAAGAA | GCTGGACTGATTGAGGGTGC |

**Table S4.** The details of antibodies used in the current study.

| Antibody | Host | Company (Cat No.) |
| --- | --- | --- |
| Anti-mouse-GAPDH | Mouse | Proteintech, USA (60004-1-Ig) |
| Anti-Vimentin | Mouse | Abcam, UK (ab8069) |
| Anti-Vimentin | Rabbit | CST, USA (#5741) |
| Anti-CLCNKA | Rabbit | Proteintech, USA (14402-1-AP) |
| Anti-AQP2 | Rabbit | Abcam, UK (ab199975) |
| Anti-E-cadherin | Mouse | Proteintech, USA (60335-1-Ig) |
| Anti-CD34 | Rabbit | Abcam, UK (ab81289) |
| Anti-Runx2 | Rabbit | CST, USA (#12556) |
| Anti-Runx2 | Mouse | Abcam, UK (ab76956) |
| Anti-OCN | Rabbit | Abcam, UK (ab133612) |
| Anti-OCN | Mouse | Abmart, China (MK47122S) |
| Anti-BMP2 | Mouse | Proteintech, USA (66383-1-Ig) |
| Anti-BMP2 | Rabbit | Abcam, UK (ab214821) |
| Anti-OMD | Rabbit | Abcam, UK (ab154249) |
| Anti-ubiquitin | Mouse | Santa Cruz Biotechnology, UAS (sc-8017) |
| Anti-SMURF1 | Rabbit | Proteintech, USA (55175-1-AP) |
| Anti-SMURF2 | Rabbit | Proteintech, USA (18038-1-AP) |
| Anti-NEDD4 | Rabbit | Abcam, UK (ab317414) |
| Anti-BMPR1A | Rabbit | Proteintech, USA (12702-1-AP) |
| Anti-BMPR1B | Rabbit | Abcam, UK (ab175385) |
| Anti-BMPR2 | Rabbit | Proteintech, USA (19087-1-AP) |
| Anti-Cre Recombinase | Rabbit | CST, USA (#15036) |
| Anti-COL1A2 | Rabbit | Abcam, UK (ab308455) |
| HRP-conjugated anti-Rabbit IgG | Goat | Proteintech, USA (SA00001-2) |
| HRP-conjugated anti-Mouse IgG | Goat | Proteintech, USA (SA00001-1) |
| Anti-Mouse IgG H&L (Alexa Fluor®488) | Goat | Abcam, UK (ab150113) |
| Anti-Rabbit IgG H&L (Alexa Fluor®488) | Goat | Abcam, UK (ab150077) |
| Anti-Mouse IgG H&L (Alexa Fluor®647) | Goat | Abcam, UK (ab150115) |
| Anti-Rabbit IgG H&L (Alexa Fluor®647) | Goat | Abcam, UK (ab150083) |

**Table S5.** Primer sequences designed for CHIP-qPCR

| Target regions | Forward primer 5′–3′ | Reverse primer 5′–3′ |
| --- | --- | --- |
| RUNX2 binding sites in *OMD* promoter (394bp) | AGGTGTTGAATAACCAAGGGAC | CACTGTGACAGGGAACTCCA |
| SMAD3 binding sites in *OMD* promoter (358bp) | TTGAGACAGTCTGGCTCTGTCG | ACTGCACTCCAGCCTGGGCAA |
| RNA Polymerase II binding sites in *GAPDH* promoter (positive control; 110bp) | Involved in the kit | Involved in the kit |

**Table S6.** Predicted binding sites for RUNX2 and SMAD3 in the *OMD* Promoter.

| TFs | Motif | Strand of *OMD* | Score | P-value | Matched sequence | Motif Site | Motif web |
| --- | --- | --- | --- | --- | --- | --- | --- |
| RUNX2 | MA0511.1 | + | 14.3 | 8.26*10^-06^ | GGAGGTT GTGGTGAG | TSS -275bp | <http://jaspar.genereg>.  net/matrix/MA0511.1 |
| SMAD3 | SMAD3_ HUMAN. H11MO.0.B | - | 12.5 | 7.72*10^-06^ | CTCAG CCTCCTG | TSS -2806bp | <http://hocomoco11.autosome>.  ru/motif/SMAD3_HUMAN.H11MO.0.B |

TF, transcription factors; TSS -, upstream of transcriptional start.

**Table S7.** Primer sequences designed for mouse genotyping.

| Gene | Forward primer 5′–3′ | Reverse primer 5′–3′ |
| --- | --- | --- |
| *Omd* (wt:135bp; flox: 200bp) | TTCAGTAACAGAAGTAGTAAGGGACAG | CACTGTAAATGCTGGGCATTCTTG |
| Cre (400bp) | TGCCACGACCAAGTGACAGCAATG | AGAGACGGAAATCCATCGCTCG |

**Table S8.** Proteomics data on IgG immune-precipitates screened by mass spectrometry (Excel file).

**Table S9.** Proteomics data on anti-OMD immune-precipitates screened by mass spectrometry (Excel file).

**Table S10.** Transcription factors with predicted binding sites in the *OMD* promoter

| Transcription factors binding to OMD promoter predicted by JASPAR and HOCOMOCO (n=127) |
| --- |
| ASCL1 ATOH1 BATF3 CEBPA E2F1 E2F4 E2F6 E2F7 EBF1 EGR1 EGR2 ELF3 ESRRA ESRRB EWSR1-FLI1 FEZF1 FOXC1 FOXC2 FOXF2 FOXI1 FOXJ2 FOXJ3 FOXM1 FOXO4 FOXP1 FOXQ1 GATA3 GATA6 HES7 HNF1B HOXB13 INSM1 IRF1 IRF3 IRF7 IRF8 KLF4 KLF5 KLF9 LHX3 MAFB MAZ MECOM MYBL1 MZF1 NEUROD1 NEUROD2 NEUROG2 NFATC3 NFKB1 NFKB2 NKX2-3 NKX3-1 NKX3-2 NR1D1 NR1H3 NR2C2 NR2E3 NR2F1 NR2F2 NR3C1 NR5A1 NR5A2 PATZ1 PAX5 PBX1 POU1F1 POU2F1 POU3F1 POU3F2 POU3F3 POU4F1 POU4F2 POU4F3 POU6F1 PRDM6 RARA REL RELA RELB RFX1 RFX2 RUNX2 RXRA RXRB RXRG SMAD3 SMARCA5 SOX10 SOX13 SOX2 SOX4 SOX8 STAT2 TAF1 TBR1 TEAD4 TFDP1 THAP1 THAP11 THRA THRB TP63 TP73 TWIST1 ZBTB17 ZBTB18 ZIM3 ZKSCAN1 ZNF121 ZNF134 ZNF140 ZNF143 ZNF24 ZNF263 ZNF320 ZNF329 ZNF354A ZNF384 ZNF394 ZNF436 ZNF502 ZNF586 ZNF768 ZNF770 ZNF816 ZSCAN22 |

JASPAR, <https://jaspar.elixir.no/>; HOCOMOCO, <https://hocomoco11.autosome.org/>.

**Supplementary Figures**

**
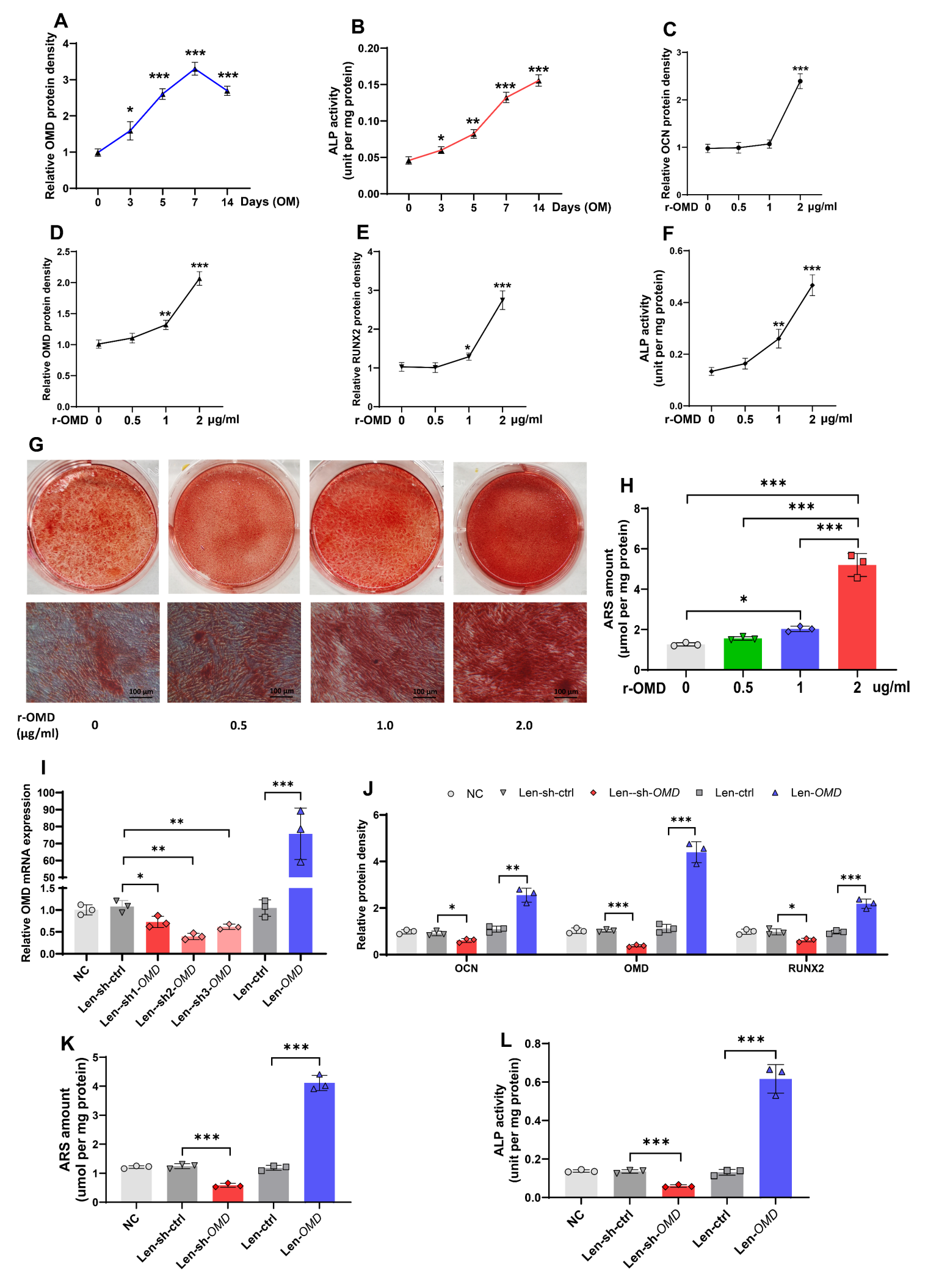
**

**Figure S1. (A)** Quantification of OMD determined by WB in hRIFs 0, 3, 5, 7, and 14 days after culture in osteogenic medium (OM); n=3. **(B)** Alkaline phosphatase (ALP) activity in hRIFs 0, 3, 5, 7, and 14 days after culture in OM; n=3. ALP activity was normalized to the total protein of cell lysate, expressed as a unit per mg of protein. **(C-E)** Quantification of OCN, OMD, and RUNX2 determined by WB in hRIFs co-cultured with recombinant human OMD protein (r-OMD); n=3. **(G-H)** Alizarin red staining was performed for hRIFs co-cultured with r-OMD in OM for 14 days, and alizarin red was quantified and normalized to the total protein of cell lysate, expressed as μmol per mg of protein; n=3. **(I)** hRIFs were transfected with recombinant lentivirus to overexpress or silence *OMD*, and qRT-PCR determined the efficiency; n=3. Len-sh2-*OMD* was chosen for silencing *OMD* in subsequent experiments due to its superior efficiency. **(J)** Quantification of OCN, OMD, and RUNX2 determined by WB in hRIFs with either overexpression or silence of *OMD*; n=3. **(K)** Alizarin red staining was performed for hRIFs with either overexpression or silence of *OMD* in OM for 14 days, and alizarin red was quantified and normalized to the total protein of cell lysate, expressed as μmol per mg of protein; n=3. **(L)** ALP activity in hRIFs with either overexpression or silence of *OMD* in OM for 14 days. ALP activity was normalized to the total protein of cell lysate, expressed as unit per mg of protein; n=3.


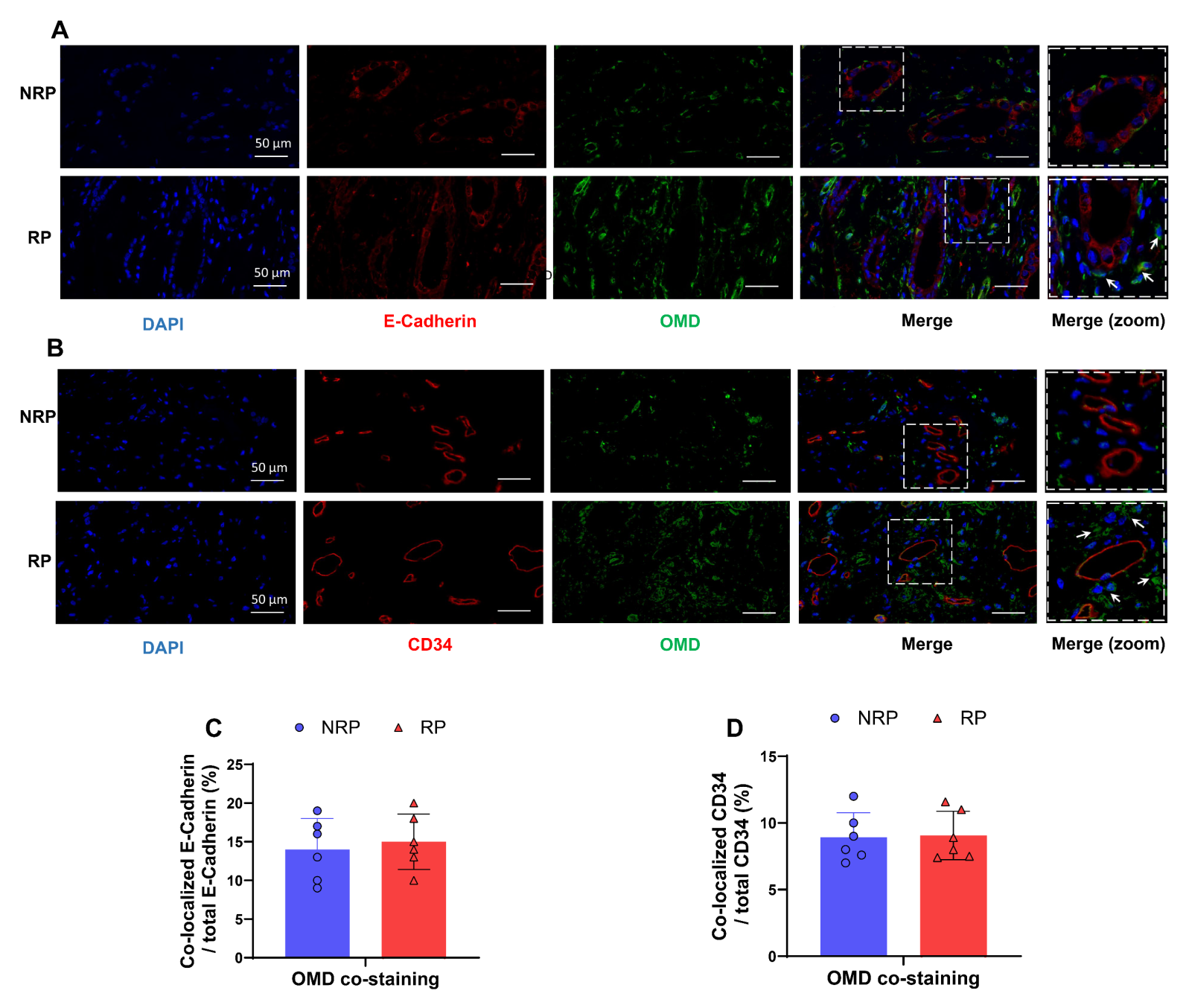


**Figure S2. (A)** Immunofluorescence co-staining for E-Cadherin and OMD in normal renal papillae (NRP; n=6) and Randall’s plaque (RP; n=6) tissues. **(B)** Immunofluorescence co-staining for CD34 and OMD in NRP (n=6) and RP (n=6) tissues. **(C)** The percentage of E-Cadherin co-localized with OMD to the total E-Cadherin; n=6. **(D)** The percentage of CD34 co-localized with OMD to the total CD34; n=6.
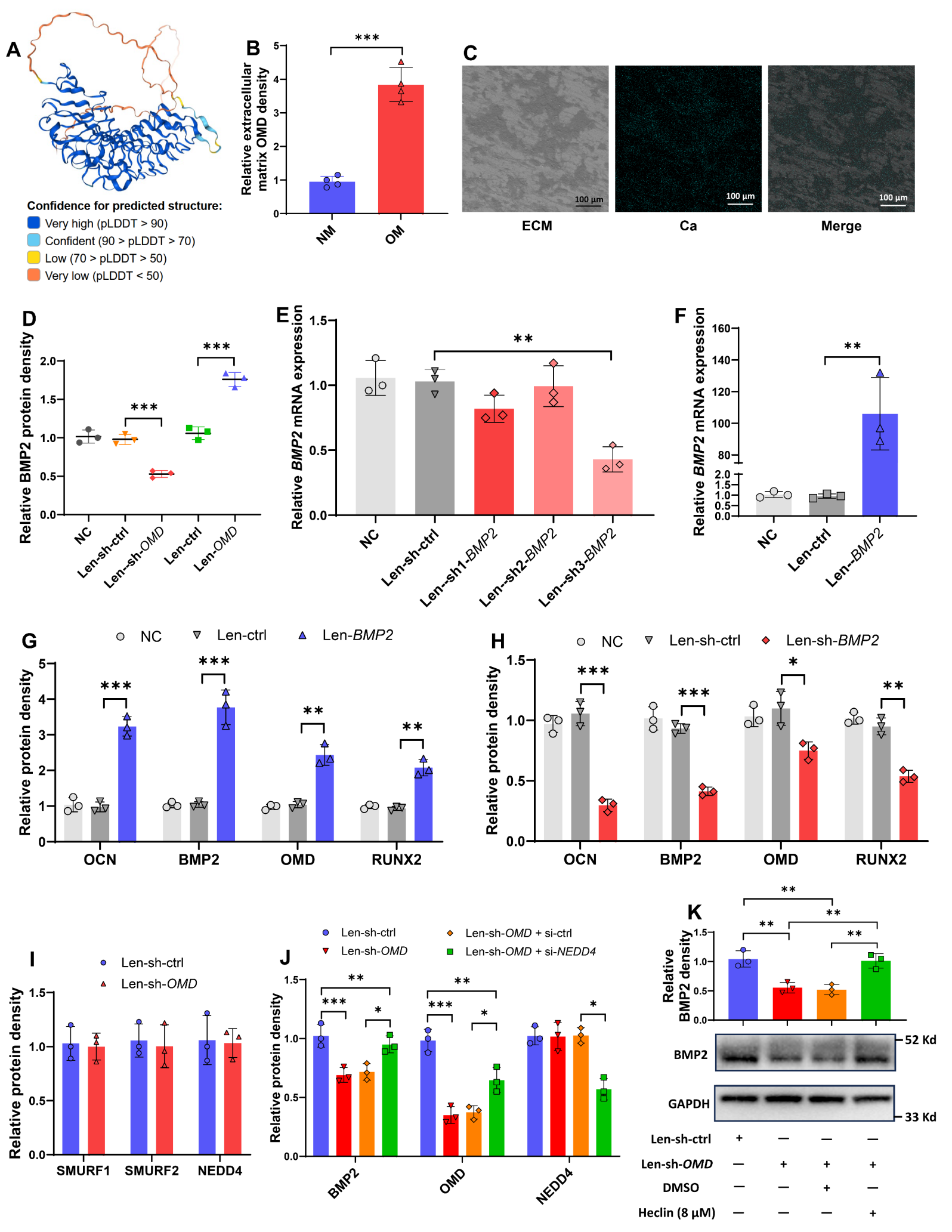


**Figure S3. (A)** 3D-structure of OMD predicted in The Human Protein Altas (<https://www.proteinatlas.org>). **(B)** After 14 days of culture in normal medium (NM) or osteogenic medium (OM), semi-quantification of OMD in extracellular matrix (ECM) determined by immunofluorescence; n=4. **(C)** Scanning electron microscopy coupled with X-ray energy dispersive spectroscopy revealed the co-localization of calcium element and ECM extracted from hRIFs. **(D)** Quantification of BMP2 determined by WB in hRIFs with either overexpression or silence of *OMD*. **(E-F)** hRIFs were transfected with recombinant lentivirus to overexpress or silence *OMD*, and qRT-PCR determined the efficiency; n=3. Len-sh3-*BMP2* was chosen for silencing *BMP2* in subsequent experiments due to its superior efficiency. **(G-H)** Quantification of OCN, BMP2, OMD, and RUNX2 determined by WB in hRIFs with either overexpression or silence of *BMP2*; n=3. **(I)** Quantification of E3 ubiquitin ligases (SMURF1; SMURF2; NEDD4) determined by WB in hRIFs transfected with Len-sh-ctrl or Len-sh-*OMD*; n=3. **(J)** Quantification of BMP2, OMD and NEDD4 determined by WB in hRIFs co-transfected with Len-sh-*OMD* and si-*NEDD4*; n=3. **(K)** After treatment with NEDD4 inhibitor (Heclin; 8μM), BMP2 was determined by WB in hRIFs transfected with Len-sh-ctrl or Len-sh-*OMD*; n=3.


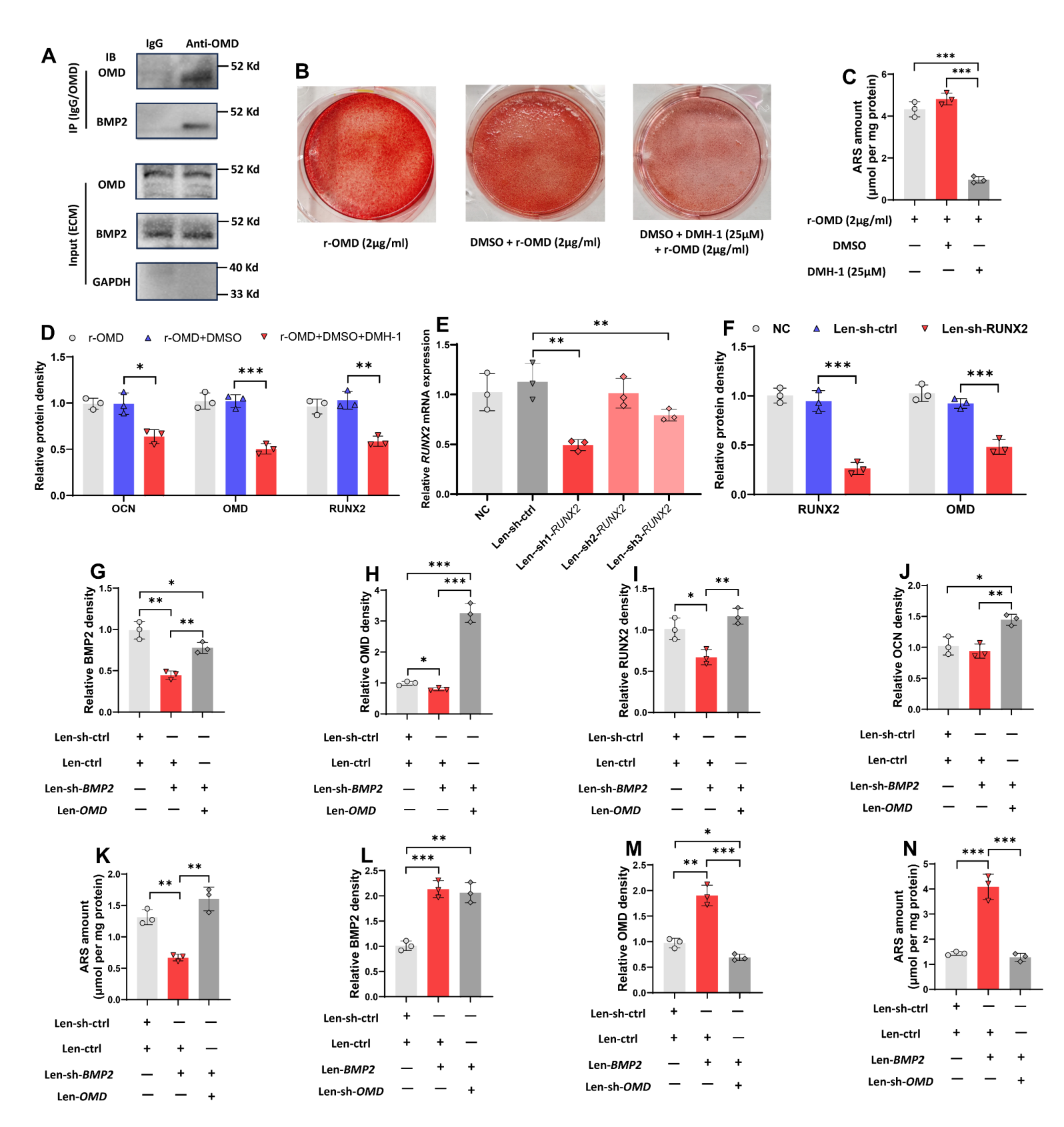


**Figure S4. (A)** Extracellular matrix (ECM) was extracted from hRIFs after 7 days of osteogenic induction. Immunoblotting (IB) for GAPDH was performed to ensure that the ECM was free of cytoplasmic protein contamination prior to lysis. Immunoprecipitation (IP) of ECM lysate was then conducted to assess the extracellular interaction between OMD and BMP2; n=3. **(B-C)** hRIFs were co-cultured with r-OMD (2μg/ml) and DMH-1 (25μM) in osteogenic medium (OM) for 14 days, and alizarin red staining (ARS) was performed; n=3. **(D)** Quantification of OCN, OMD, and RUNX2 determined by WB in hRIFs co-cultured with r-OMD and DMH-1 in OM for 7 days; n=3. **(E)** hRIFs were transfected with recombinant lentivirus to silence *RUNX2*, and qRT-PCR determined the efficiency; n=3. Len-sh1-*RUNX2* was chosen for silencing *RUNX2* in subsequent experiments due to its superior efficiency. **(F)** Quantification of OMD and RUNX2 determined by WB in hRIFs transfected with Len-sh-*RUNX2* after osteogenic induction for 7 days; n=3. **(G-J)** hRIFs were co-transfected with Len-sh-*BMP2* and either Len-ctrl or Len-*OMD*, and then induced with osteogenic medium for 7 days. ImageJ was utilized to quantify the relative expression levels of BMP2, OMD, RUNX2, and OCN to GAPDH, determined by WB; n=3. **(K)** Co-transfected hRIFs were induced with osteogenic medium for 14 days, and then ARS was performed. Alizarin red was quantified and normalized to the total protein of cell lysate; n=3. **(L-M)** hRIFs were co-transfected with Len-*BMP2* and either Len-ctrl or Len-sh-*OMD*, and then induced with osteogenic medium for 7 days. ImageJ was utilized to quantify the relative expression levels of BMP2 and OMD determined by WB; n=3. **(N)** Co-transfected hRIFs were induced with osteogenic medium for 14 days, and then ARS was performed. Alizarin red was quantified and normalized to the total protein of cell lysate; n=3.

**
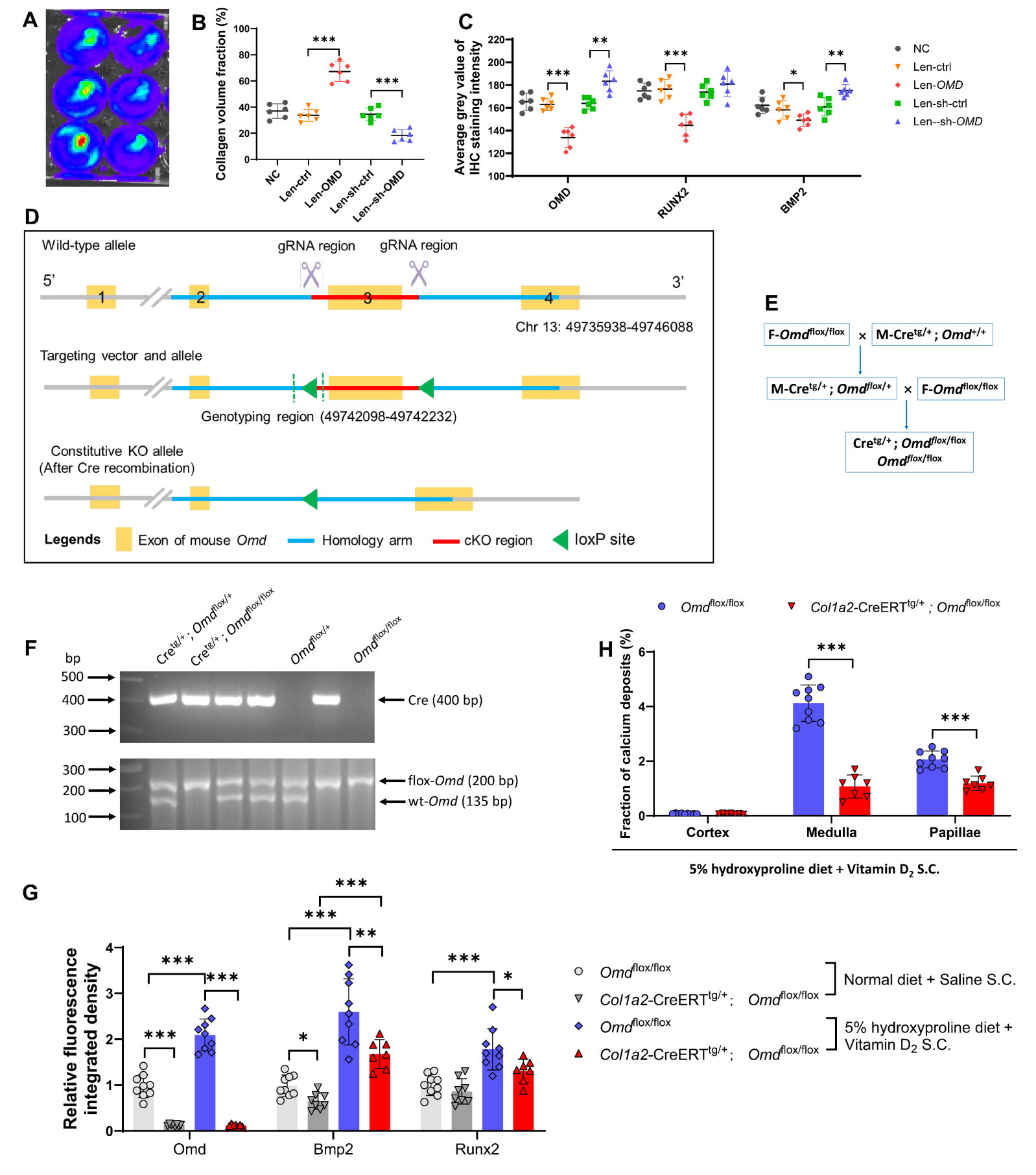
**

**Figure S5. (A)** Fluorescence was detected in luciferase-expressing hRIFs *in vitro* following injection of D-Luciferin. **(B)** The collagen volume fraction was quantified by measuring the blue-stained collagen fibers in Masson's trichrome staining of subcutaneous implants using ImageJ. The stained area within hydroxyapatite scaffolds was excluded from the analysis; n=6 for each group. **(C)** The immunohistochemistry (IHC) staining intensity (OMD; RUNX2; BMP2) of subcutaneous implants was quantified by average grey value using ImageJ; n=6 for each group. The average grey value ranged from 0 to 254, where a black, dark-stained area was assigned a grey value of 0, and a white, unstained area was assigned a grey value of 254. This setup resulted in an inverse correlation between the average grey value and staining intensity. **(D)** A schematic diagram illustrating generation of germline *Omd^flox/flox^* mice. Exon 3 was selected as the conditional knockout region (cKO region), and primer positions were shown. **(E)** The mating scheme used to target ablation of *Omd* in postnatal renal interstitial fibroblasts with tamoxifen-inducible Cre under the control of the proα2(I) collagen (*Col1a2*) promoter (*Col1a2-CreERT^tg/+^*; *Omd^flox/flo^*^x^). **(F)** Genotyping PCR products of littermates from the mating of *Col1a2-CreERT^tg/+^*; *Omd^flox/flox^* with *Omd^flox/flox^*. **(G)** *Omd^flox/flox^* mice and *Col1a2-CreERT^tg/+^*; *Omd^flox/flox^* mice either underwent induction of CaOx nephrocalcinosis or were assigned to a control group. Immunofluorescence co-staining of Omd and either Bmp2 or Runx2 was performed in Col1a2-marked renal interstitial fibroblasts. Semi-quantitative analysis of fluorescence cumulative intensity was performed using ImageJ. The fluorescence cumulative intensity for each image was calculated as the fluorescence expression area multiplied by the fluorescence expression grayscale value. **(H)** Von-Kossa staining was performed to assess calcium deposits in renal cortex, medulla, and papillae. The fraction of Von-Kossa staining area was analyzed using ImageJ. Normal diet + Saline S.C. group (*Omd^flox/flox^* mice, n=9; *Col1a2-CreERT^tg/+^*; *Omd^flox/flox^* mice, n=8); 5% hydroxyproline diet + Vitamin D_2_ S.C. group (*Omd^flox/flox^* mice, n=9; *Col1a2-CreERT^tg/+^*; *Omd^flox/flox^* mice, n=7). S.C., subcutaneous injection.


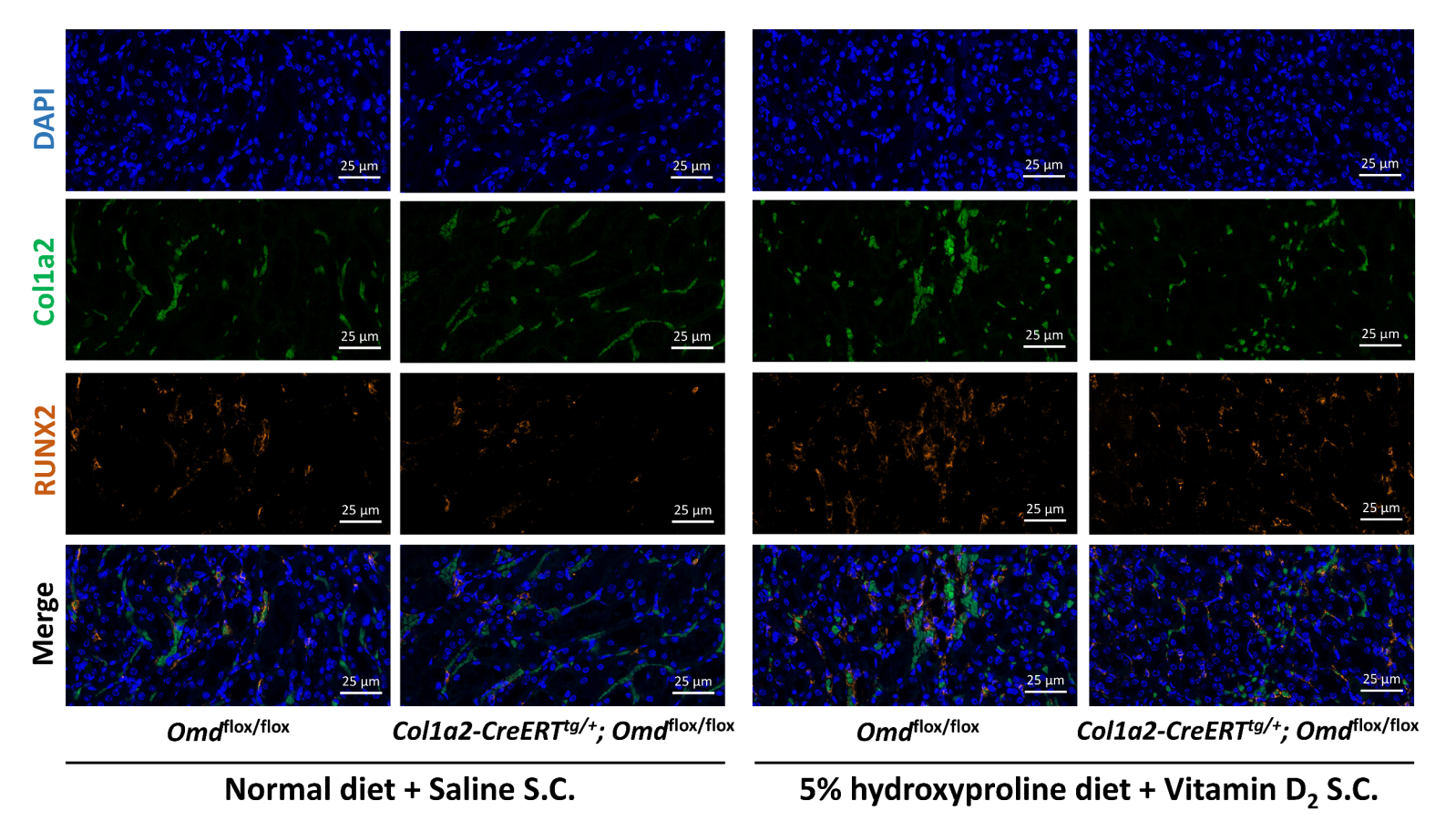


**Figure S6.** *Omd^flox/flox^* mice and *Col1a2-CreERT^tg/+^*; *Omd^flox/flox^* mice either underwent induction of CaOx nephrocalcinosis or were assigned to a control group. Immunofluorescence staining of Runx2 was performed in Col1a2-marked renal interstitial fibroblasts. Normal diet + Saline S.C. group (*Omd^flox/flox^* mice, n=9; *Col1a2-CreERT^tg/+^*; *Omd^flox/flox^* mice, n=8); 5% hydroxyproline diet + Vitamin D_2_ S.C. group (*Omd^flox/flox^* mice, n=9; *Col1a2-CreERT^tg/+^*; *Omd^flox/flox^* mice, n=7).

**References**

[1] Z. Zhu, F. Huang, W. Xia, H. Zeng, M. Gao, Y. Li, F. Zeng, C. He, J. Chen, Z. Chen, Y. Li, Y. Cui, H. Chen, *Front Cell Dev Biol* **2020**, *8*, 596363.

[2] J.R. van Beijnum, M. Rousch, K. Castermans, E. van der Linden, A.W. Griffioen, *Nat Protoc* **2008**, *3*, 1085.

[3] P.C. Baer, W.A. Nockher, W. Haase, J.E. Scherberich, *Kidney Int* **1997**, *52*, 1321.

[4] Y. Huang, Y. Zheng, L. Jia, W. Li, *Stem Cells* **2015**, *33*, 3481.

[5] C.A. Gregory, W.G. Gunn, A. Peister, D.J. Prockop, *Anal Biochem* **2004**, *329*, 77.

[6] Z. Zhu, S. Ruan, Y. Jiang, F. Huang, W. Xia, J. Chen, Y. Cui, C. He, F. Zeng, Y. Li, Z. Chen, H. Chen, *Cell Mol Life Sci* **2021**, *78*, 7831.

[7] G. Sarila, T. Bao, S.A. Abeydeera, R. Li, B. Mell, B. Joe, A. Catubig, J. Hutson, *J Pediatr Surg* **2020**, *55*, 1952.

[8] H.M. Pazos, W.S. Costa, F.J. Sampaio, L.A. Favorito, *Cells Tissues Organs* **2010**, *191*, 422.

[9] J. Shu, G.E. Dolman, J. Duan, G. Qiu, M. Ilyas, *Biomed Eng Online* **2016**, *15*, 46.

[10] A.C. Ruifrok,D.A. Johnston, *Anal Quant Cytol Histol* **2001**, *23*, 291.
